# Iterative Structure-Based Peptide-Like Inhibitor Design against the Botulinum Neurotoxin Serotype A

**DOI:** 10.1371/journal.pone.0011378

**Published:** 2010-06-30

**Authors:** Jorge E. Zuniga, Jared T. Hammill, Omri Drory, Jonathan E. Nuss, James C. Burnett, Rick Gussio, Peter Wipf, Sina Bavari, Axel T. Brunger

**Affiliations:** 1 Howard Hughes Medical Institute and Departments of Molecular and Cellular Physiology, Neurology and Neurological Science, Structural Biology, and Photon Science, Stanford University, Stanford, California, United States of America; 2 Center for Chemical Methodologies and Library Development, University of Pittsburgh, Pittsburgh, Pennsylvania, United States of America; 3 Division of Bacteriology, Department of Immunology, Target Identification, and Translational Research, United States Army Medical Research Institute of Infectious Diseases, Frederick, Maryland, United States of America; 4 Target Structure-Based Drug Discovery Group, National Cancer Institute at Frederick, Frederick, Maryland, United States of America; 5 Information Technology Branch, Developmental Therapeutics Program, National Cancer Institute at Frederick, Frederick, Maryland, United States of America; Hospital Vall d'Hebron, Spain

## Abstract

The botulinum neurotoxin serotype A light chain (BoNT/A LC) protease is the catalytic component responsible for the neuroparalysis that is characteristic of the disease state botulism. Three related peptide-like molecules (PLMs) were designed using previous information from co-crystal structures, synthesized, and assayed for *in vitro* inhibition against BoNT/A LC. Our results indicate these PLMS are competitive inhibitors of the BoNT/A LC protease and their K_i_ values are in the nM-range. A co-crystal structure for one of these inhibitors was determined and reveals that the PLM, in accord with the goals of our design strategy, simultaneously involves both ionic interactions via its P1 residue and hydrophobic contacts by means of an aromatic group in the P2′ position. The PLM adopts a helical conformation similar to previously determined co-crystal structures of PLMs, although there are also major differences to these other structures such as contacts with specific BoNT/A LC residues. Our structure further demonstrates the remarkable plasticity of the substrate binding cleft of the BoNT/A LC protease and provides a paradigm for iterative structure-based design and development of BoNT/A LC inhibitors.

## Introduction

Botulinum neurotoxins (BoNTs), secreted by *Clostridium botulinum* [1], provide invaluable treatments for a range of medical conditions [2], [3], [4], [5], [6], [7], [8], [9], [10], [11] and cosmetic purposes [12], [13], [14], [15], [16]. Paradoxically, BoNTs are also the most potent biological toxins known by causing the disease state botulism. As a result, these enzymes are classified as category A biothreat agents by the Centers for Disease Control and Prevention (http://emergency.cdc.gov/agent/agentlist-category.asp), with the clandestine contamination of liquids and/or food stuffs being plausible scenarios [17], [18].

The seven known BoNT serotypes are designated A – G. Post secretion, they undergo proteolytic processing to provide the bioactive (i.e., poisonous) holotoxin [1]. The holotoxin is composed of a 100 kDa heavy chain (HC) subunit and a 50 kDa light chain (LC) subunit; these two components are tethered by a disulfide bridge [1], [19], [20]. Mechanistically, the HC binds to specific motor neuron receptors and induces endosomal internalization [1]. The LC (BoNT/LC) is a zinc (Zn)(II) metalloprotease that is released from the holotoxin into the neuronal cytosol [1]. Once inside the neuronal cytosol, the LC cleaves specific peptide bonds (depending on the serotype) of proteins composing the neuronal SNARE complex: the synaptosomal-associated protein of 25 kDa (SNAP-25), the vesicle-associated membrane protein (VAMP), also referred to as synaptobrevin, and syntaxin [1], [21]. Botulinum neurotoxin serotypes A, C, and E cleave SNAP-25 [22], [23], [24]; serotypes B, D, F, and G cleave VAMP [25], [26], [27], [28], and BoNT serotype C also cleaves syntaxin [23]. The BoNT/LC mediated proteolytic cleavage of any one of the three SNARE proteins prevents acetylcholine-filled vesicles in the neuron from fusing with the active zone at the synaptic cleft [1]. This inhibits the transmission of motor nerve impulses, and as indicated above, results in the flaccid paralysis that is characteristic of botulism [29].

At present, the only treatments available for BoNT intoxication involve antitoxin administration [1], followed by critical care mechanical respiration. However, this treatment would not be practical for treating even a modest number of poisoned individuals: antitoxin administration is ineffective after BoNT internalization (and it is likely that victims would seek medical attention only after the paralysis manifestation). Critical care mechanical respiration is costly and the small number of medical facilities in the U.S. equipped with such devices would more than likely be overwhelmed. Thus, there is an urgent need for the development of small-molecule inhibitors of BoNT LCs.

Of the seven BoNT serotype LCs, the BoNT serotype A LC (BoNT/A LC) possesses the longest duration of action in the neuronal cytosol [30]. Hence, there continues to be a significant effort to identify and develop both peptidic and small, drug-like molecule inhibitors [1] of this particular serotype LC. Previously, we identified and developed BoNT/A LC inhibitors involving the simultaneous identification, design, and generation of both small molecule, non-peptidic, inhibitors (SMNPIs) [29], [31], [32], [33], [34] and peptide-like molecules (PLMs) [35], [36]. Such PLM design complements SMNPI development, as BoNT/A LC:PLM co-crystal structures form the bases for: 1) the design and synthesis of more potent, drug-like peptidomimetics, 2) the rational, structure-based modification of existing SMNPIs to improve inhibitory efficacies, and 3) the discovery and development of novel SMNPIs via database mining (employing PLM binding modes as search query templates). For example, the conformation and chemical contacts of a PLM bound to the BoNT/A LC can be used to generate three-dimensional (3D) search queries to discover new SMNPI chemotypes via the database mining of virtual small molecule libraries.

**Figure 1:**
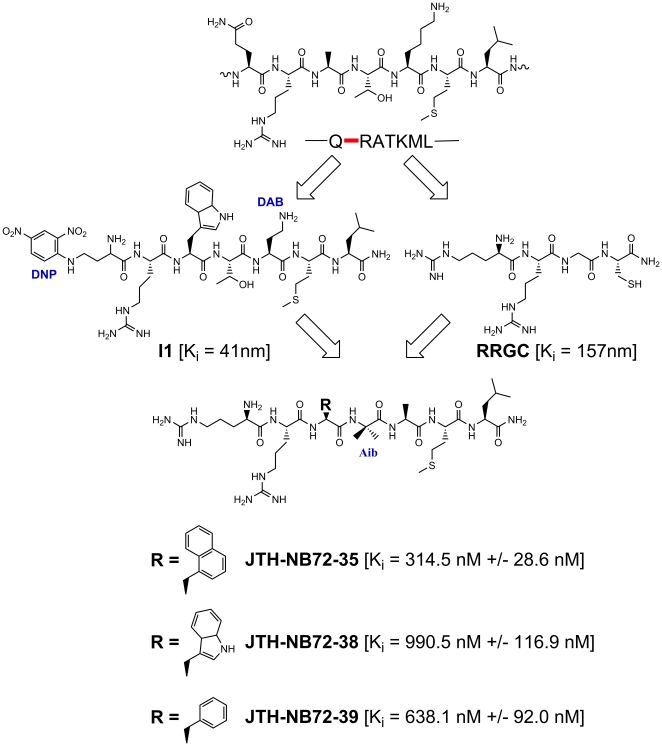
PLM structures, K_i_ values, and design strategy. From top to bottom, schematic representation of the SNAP-25 P1-P6′ segment, with the substrate scissile bond indicated in red; representation of the **I1** and RRGC PLMs and their corresponding inhibition constants; schematic representation of the design strategy for the **JTH-NB72** PLMs, showing the incorporation of the Arg and the aromatic sidechain (R) in the P1 and the P2′ positions, respectively. The aromatic structures tested in the three **JTH-NB72** PLMs are indicated, together with their corresponding K_i_ values and standard deviations (these values were calculated as the average of three different measurements).

Here, we describe three new PLMs (Figure 1) that were designed to explore the BoNT/A LC substrate cleft based on the inhibitor-protease interactions found in a previously published co-crystal structure of BoNT/A LC with the inhibitor **I1** [35]. The three new PLMs possess K_i_ values in the nM range which, together with **I1**, place them among the most potent BoNT/A LC inhibitors characterized to date. One of the PLMs, **JTH-NB72-39**, was co-crystallized in complex with the BoNT/A LC protease, confirming the interactions aimed by our design strategy while revealing new, unforeseen inhibitor:enzyme contacts that will preface future studies to design more potent PLM and SMNPI inhibitors.

## Results and Discussion

### Inhibitor Design

Previously, we reported several nanomolar (nM)-range PLM inhibitors resembling the cleavage site sequence of SNAP-25 [35]. The seven-residue P1-P6′ sequence QRATKML (residue positions 197–203 of human SNAP-25) was used to design the PLMs. Of these, a co-crystal structure of the BoNT/A LC with PLM **I1** (Figure 1) was determined and its binding contacts and mechanism of inhibition (with respect to the LC's active site) were studied in detail [35]. Based on a general design strategy (Figure 1) and the BoNT/A LC:**I1** co-crystal structure, we attempted to increase the inhibitory potency of the PLM **I1** ‘template’ by replacing and incorporating different components to increase both ionic and hydrophobic contacts with residues in the enzymes's binding cleft, and to stabilize the 3_10_ helical conformation of **I1**. We hypothesized that such conformational stabilization of the otherwise flexible inhibitor would decrease the binding entropy of the resulting inhibitors, and thus increase affinity. Along these lines, we restrained the conformation of **I1**, as it is bound to the BoNT/A LC [35], and thus attempted to reduce the inhibitor's binding entropy, by introducing an aminoisobutyric acid (Aib) residue (Figure 1), which is known to favor the type II' β-turn repeat in a 3_10_ helix [37], [38]. In addition, we replaced the redox-active DNP-DAP functional group of **I1** [35] with an Arg residue, which, as Kumaran *et al*. [39], [40] demonstrated with co-crystal structures, provides direct electrostatic contacts with anionic residues in the BoNT/A LC substrate cleft (in contrast to the DNP-DAP residue of **I1** [35]). Furthermore, we increased the hydrophobic nature of the C-terminus of **I1** by replacing the DAB residue [35] with an Ala residue (as found in the P4′ position of SNAP-25). Finally, we allowed for combinatorial exploration of the aromatic hydrophobic interactions of the **I1** Trp residue (Figure 1), mainly by substituting this position with two sterically and electronically diverse benzyl and naphthylene methylene substituents. The resulting PLM designs **JTH-NB72-35**, **JTH-NB72-38**, and **JTH-NB72-39** are shown in Figure 1.

### Inhibitor Synthesis

The synthesis of the PLMs used manual microwave assisted solid phase peptide synthesis using Fmoc protected amino acids and Rink amide SS resin. After swelling the resin in dichloromethane solvent for 30 min, a stepwise synthesis was initiated by removal of the Fmoc protecting group from the Rink amide resin with a solution of 20% piperidine in DMF. The newly formed free amine was then coupled to the activated, protected amino acid corresponding to the C-terminus of the desired PLM. Initial attempts to activate the protected amino acids for coupling using PyBop and HOBt provided unsatisfactory yields; however, a brief screening of activating agents revealed that Goodman's reagent (DEPBT) provided the desired PLMs in good yield [41], [42]. With the first amino acid successfully coupled to the Rink resin, the Fmoc group was again removed, and the subsequent, activated, amino acid was coupled to the freshly deprotected peptide chain. This process of deprotection and coupling was repeated until the amide-terminal residue of the desired PLM was appended. Following the final Fmoc deprotection, the PLMs were cleaved from the solid support by stirring in a modified version of Reagent K (87.5% TFA, 3.6% thioanisole, 2.3% EDT, 3.7% phenol, 1.8% H_2_O, 1.1% triisopropylsilane) for 2 h at RT. Precipitation with diethyl ether provided crude products, which were subsequently purified by preparative RP HPLC (34–42% yield).

### *In Vitro* Inhibition

Using the methods described below, we obtained K_i_ values in the nM range for the **JTH-NB72-35**, **JTH-NB72-38**, and **JTH-NB72-39** PLMs (Figure 1), although none of them were as potent as **I1**. Therefore, co-crystallization experiments were conducted in order to collect any structural information that might explain this unexpected result.

### Co-crystal Structure of PLM JTH-NB72-39 in complex with BoNT/A LC

**Figure 2:**
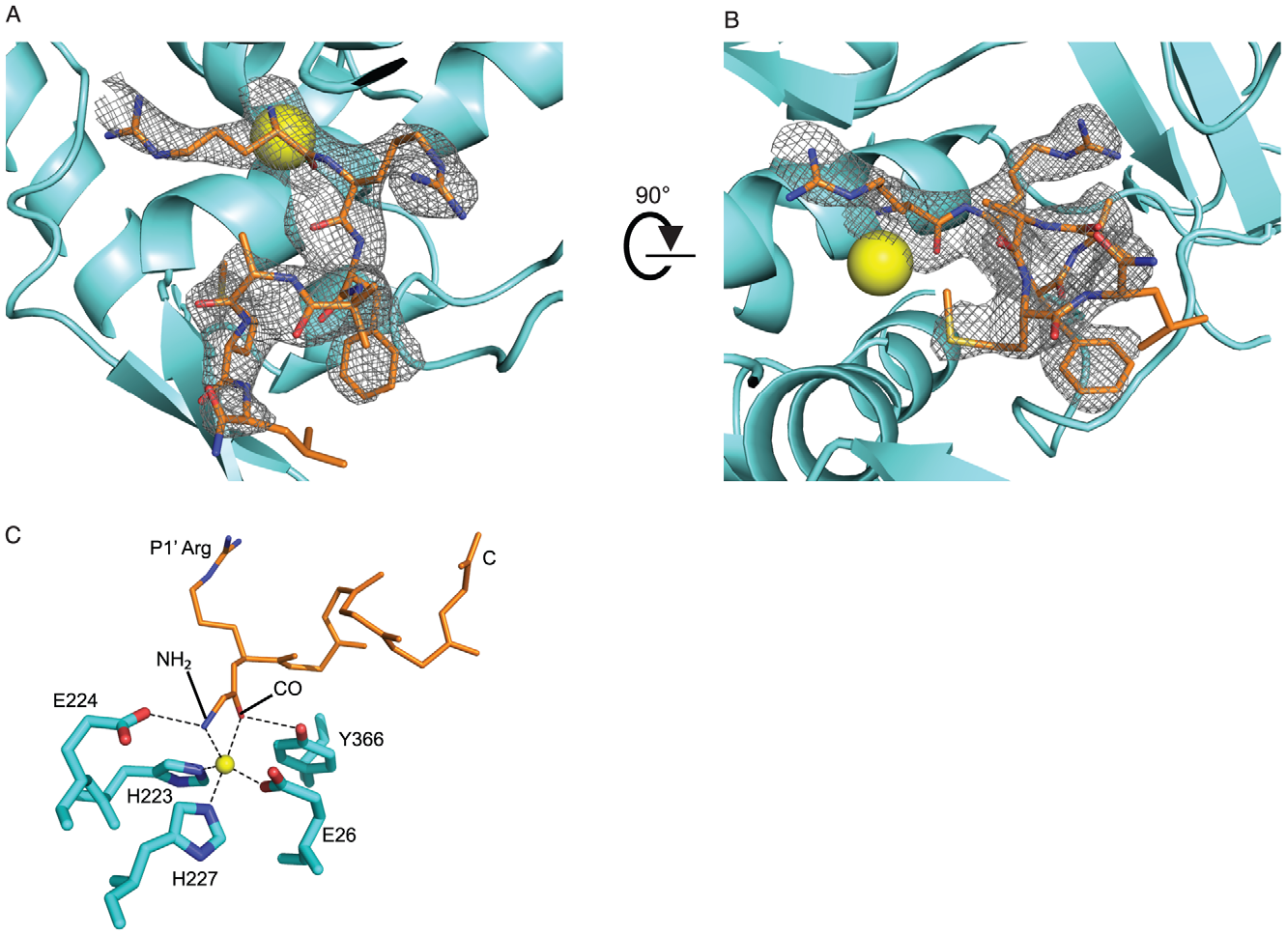
Initial electron density for the JTH-NB72-39 inhibitor and inhibition of the BoNT/A LC ‘catalytic engine’. **A.** View of the initial σ_A_-weighted F_o_-F_c_ difference electron-density map contoured at 2.0 σ (grey mesh) around the inhibitor-binding site, and overlaid with the refined model of the complex (**JTH-NB72-39** is depicted in orange sticks, the Zn(II) atom as a yellow sphere, and the BoNT/A LC in cyan ribbon representation). The map was computed with phases calculated prior to the inclusion of **JTH-NB72-39** (i.e. it is a model-bias free map). For the PLM nitrogen, oxygen, and sulfur atoms are colored blue, red, and yellow, respectively. **B.** The same as A, but visualized from a different angle. **C.** Inhibiting interactions of **JTH-NB72-39**. BoNT/A LC residues are displayed as cyan sticks, and the **JTH-NB72-39** backbone is shown as thin, orange sticks. Only the P1′ Arg side chain of the inhibitor is shown as reference. Interactions between BoNT/A LC and **JTH-NB72-39** are represented by dashed lines. The identity of the residues is indicated. The P1 amino and carbonyl groups are indicated by NH_2_ and CO, respectively. The C-terminus of the inhibitor is indicated by the letter C. The Zn(II) atom is represented as a yellow sphere.

Of the co-crystallization experiments conducted with the three PLMs**,** only BoNT/A LC:**JTH-NB72-39** produced diffracting crystals. We obtained a co-crystal structure of this complex at 2.4 Å resolution (Table 1). The structure was determined by molecular replacement using the structure of BoNT/A LC as the search model (PDB reference code 3DSE [35]), but omitting the inhibitor coordinates, water molecules, and other ligands (i.e., Zn(II) and Ni(II) ions) from the search model[35]. Significant electron density for the PLM emerged next to the catalytic Zn(II) around the binding cleft defined by loops 70, 250 and 370 in the LC protease (Figure 2).

**Table 1:** X-ray data collection and refinement.

Space group	P2_1_2_1_2
a, b, c (Å)	56.1, 189.6, 41.51
Resolution (Å)	45–2.4 (2.47–2.4)
Unique reflections	16177
Redundancy	5.5 (5.1)
Completeness (%)	93.3% (77.4%)
I/σ	33.8 (5.1)
R_sym_ (%)	7.1% (32.6%)
R_cryst_/R_free_	18.31%/23.08%
No. atoms	
BoNT/A LC	3179
JTH-NB72-39	61
Ni	1
Zn	1
Water	117
Average thermal (B) factor	
BoNT/A LC	42.30 Å^2^
JTH-NB72-39	49.50 Å^2^
Ni	43.82 Å^2^
Zn	32.14 Å^2^
Water	60.4 Å^2^
R.m.s. deviations	
Average bond length deviation	0.004 Å
Average bond angle deviation	0.802 °

### Binding interactions between PLM JTH-NB72-39 and the BoNT/A LC

The electron density for the first six residues of the PLM inhibitor is well-defined (i.e., visible at a contour level of 2.0 σ in the Fo-Fc difference electron density map), but is weaker for the last Leu residue. As discussed in detail below, most of the specific interactions observed between **JTH-NB72-39** and the BoNT/A LC are mediated by the first four residues of the PLM. Briefly, **JTH-NB72-39** also possesses the electrostatic contacts reported for the RRGC, RRGI, RRGM, and RRGL tetrapeptides, as well as for the RRATKM PLM. Moreover, our design resulted in some of the same hydrophobic interactions previously observed between **I1** and BoNT/A LC [35], but to a lesser degree.

**Figure 3:**
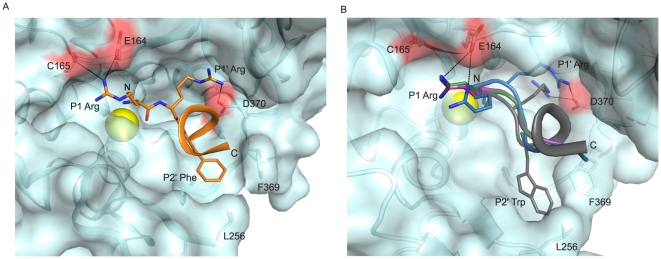
The binding of JTH-NB72-39 in the BoNT/A LC substrate cleft. **A.** Panoramic view of **JTH-NB72-39** in the binding cleft of the BoNT/A LC. **JTH-NB72-39** is displayed in orange sticks (for selected residues only) and ribbon representation, with its directionality indicated by its N and C termini. **B.** Superposition of RRGI (purple), RRGL (pink), RRGM (magenta), RRGC (green), RRATKM (blue), and **I1** (grey) PLM inhibitors in the binding cleft of the BoNT/A LC. In both panels, selected BoNT/A LC residues are shown in cyan stick and surface representation, and the nitrogen and oxygen atoms of all inhibitors are colored blue and red, respectively. The coordinates for the BoNT/A LC are those of the **JTH-NB72-39**-bound (panel A) and the **I1**-bound (panel B) complexes. The Zn(II) atom is displayed as a yellow sphere in both panels. Negatively-charged patches in the BoNT/A LC surface involved in ionic contacts (black dashes) are displayed as red surface.

**Figure 4:**
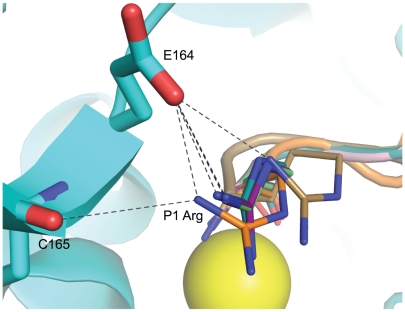
Ionic interactions for the P1 residue of PLMs. Close-up views of the P1 Arg residues of PLMs RRGI (purple carbons), RRGL (pink carbons), RRGM (magenta carbons), RRGC (pale green carbons), RRATKM (tan carbons), and **JTH-NB72-39** (orange carbons). BoNT/A LC structures in complex with the PLM inhibitors indicated above were superposed. BoNT/A LC residues are depicted in larger stick (with cyan carbons and backbone) and are taken from the coordinates of the BoNT/A LC:**JTH-NB72-39** complex. The Zn(II) ion is shown as a yellow sphere. Nitrogen and oxygen atoms are shown in blue and red, respectively.

The carbonyl oxygen of the **JTH-NB72-39** P1 residue (Arg) coordinates the enzyme's catalytic Zn(II) ion (distance is 2.4 Å) and also engages in a hydrogen bond with the hydroxyl group of residue Tyr 366 (which is known to directly stabilize the tetrahedral intermediate formed during SNAP-25 catalysis [43]), while the amino terminal group of the P1 Arg also coordinates the enzyme's catalytic Zn(II) (Figure 2C). Furthermore, the conformation of the backbone atoms of this residue is determined, in part, by electrostatic interactions between its side-chain and BoNT/A LC residues. Specifically, and in contrast to **I1**, the P1 Arg of the inhibitor engages, via a salt bridge, with the LC's Glu 164 residue while it also shares a hydrogen bond with the carbonyl oxygen of the LC's Cys 165 residue (Figures 3A and 4). A similar ionic contact with Glu 164 has been proposed for Gln 197 in the P1 position of SNAP-25 substrate during the formation of the enzyme:substrate complex [44], [45]. Moreover, the orientation of the **JTH-NB72-39** P1 Arg resembles that of the P1 Arg residues found in previously reported tetrameric inhibitors RRGI, RRGC, RRGM, and RRGL and RRATKM [40] (Figure 3B), but is in closer contact with the enzyme's Glu 164, and it is the only PLM that interacts with Cys 165 of the BoNT/A LC (Figure 4). In contrast to the binding of the P1 DNP-DAP component of **I1** [35], the **JTH-NB72-39** P1 Arg does not interact with BoNT/A LC residue Ser 259.

In the P1′ position of **JTH-NB72-39** there is a second Arg residue (Figure 3). In SNAP-25, a corresponding P1′ Arg plays a key role in facilitating hydrolysis by specifically interacting with Asp 370 in the S1′ pocket[46]. This native interaction is also exploited by all structurally known peptidic inhibitors, as all BoNT/A LC:PLM co-crystal structures reported to date posses an Arg residue at the P1′ position which provides optimal binding in the enzyme's S1′ pocket [35], [39], [40]. This Arg side chain adopts one of two rotamers, and both conformations are stabilized by the formation of a salt bridge with the side-chain carboxylate of BoNT/A LC residue Asp 370 (Figure 3). The conformation of the side-chain of the P1′ Arg of **JTH-NB72-39** closely resembles that observed for the corresponding P1′ Arg residue in PLMs RRGI, RRGL, and RRATKM [39], [40]. Additionally, the guanidinium group of the P1′ Arg engages in a cation-π interaction with Phe 194 of the LC's substrate cleft (not shown). This is a contact that is consistently observed in other BoNT/A LC:PLM co-crystal structures, and mutations of Phe 194 have been reported to diminish the catalytic efficiency of the BoNT/A LC by ∼100-fold [47]. Overall, the observed P1′-S1′ Arg:Asp 370/Phe 194 interactions appear to be key for general PLM inhibitory potency.

**Figure 5:**
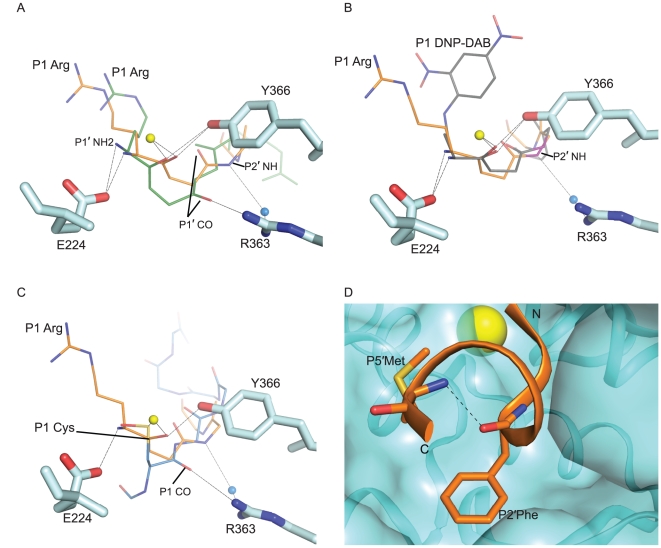
Inhibitory interactions of PLMs. Superposition of the structures of the BoNT/A LC complexes with inhibitors **JTH-NB72-39** (orange carbons) and: RRATKM (light blue carbons, panel **A**), **I1** (grey carbons, panel **B**), and CRATKML (light blue, panel **C**). BoNT/A LC residues are shown in cyan stick representation, with oxygen and nitrogen atoms colored red and blue, respectively. The Zn(II) ion and water molecule are displayed as yellow and blue spheres, respectively. The amino terminal groups of all inhibitors are labeled as NH_2_. Backbone amide groups in the P2′ position of **I1** and **JTH-NB72-39** are displayed as purple sticks. Dashed lines indicate intermolecular contacts between PLMs and the BoNT/A LC. **D.** Stick representation of the P2′ Phe and P5′ Met residues of the **JTH-NB72-39** inhibitor in its bound conformation. The intramolecular H-bond between the P2′ Phe carbonyl and the P5′ Met amide groups is indicated as a black dash line. **JTH-NB72-39** is displayed with orange carbons and ribbon; BoNT/A LC secondary structure and surface representation is colored cyan. Nitrogen, carbon, and sulfur atoms are blue, red and yellow, respectively. The Zn(II) atom is displayed as a yellow sphere.

While looking for additional contacts further down the sequence of the **JTH-NB72-39** PLM, we identified an interaction never observed before for any other BoNT/A LC inhibitor. The amide nitrogen of the **JTH-NB72-39** P2′ Phe residue engages in a water mediated interaction with the guanidinium group of BoNT/A LC residue Arg 363 (Figure 5A–C). In other BoNT/A LC:PLM complexes, such as RRATKM, and those of the tetrameric peptides RRGI, RRGL, RRGM, and RRGC [39], [40], it is the carbonyl oxygen of the PLM's P1′ that directly interacts with the enzyme's Arg 363 side-chain guanidinium group (Figure 5A). For **JTH-NB72-39**, this carbonyl group is rotated 180° relative to its orientation in the tetrameric peptides (Figure 5A). However, by virtue of this water-mediated interaction with Arg 363, the **JTH-NB72-39** P2′ Phe amide nitrogen replaces this direct interaction observed for other PLMs [39], [40]. This is relevant, as the BoNT/A LC Arg 363 is proposed to be critical for the binding and hydrolysis of the SNAP-25 substrate, presumably by maintaining proper geometry and charge distribution around the active site; mutation of this residue results in a 80-fold decrease of the catalytic rate of SNAP25 hydrolysis by BoNT/A LC [40], [43]. In addition, the side chain rotamers of Arg 363 are similar for all BoNT/A LC:PLM complexes, but differ from the rotamer observed in the unbound form of BoNT/LC, indicating that Arg 363 undergoes significant conformational changes upon PLM and substrate binding. This water-mediated contact between the **JTH-NB72-39** and Arg 363 is not observed in the **I1**-bound BoNT/A LC complex (Figure 5B).

**Figure 6:**
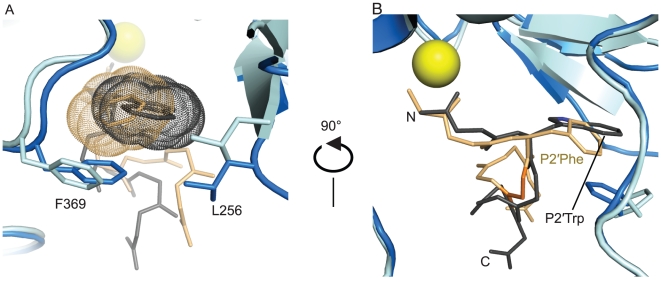
Nonpolar interactions of P2′ residues of PLMs. Superposition of the P2′ Phe of the **JTH-NB72-39** (tan sticks) and the P2′ Trp of **I1** (grey sticks) in complex with BoNT/A LC. The van der Waals surfaces (dots) of the side-chain atoms of the P2′ Phe and P2′ Trp illustrate the steric effect of these aromatic moieties. BoNT/A LC residues are colored cyan and blue for the **JTH-NB72-39**-bound and the **I1**-bound models, respectively. Phe369 and Leu256 of the BoNT/A LC are labeled with their one-letter code and number in the BoNT/A LC sequence, and their side chains are displayed in stick representation. **B**. A side view of the interactions shown in Panel A. The backbone atoms of the P3′Aib residue of **JTH-NB72-39** are colored orange. The Zn(II) ion is displayed as a yellow sphere in both panels.

Another novel interaction observed in this position of the PLM is a stabilizing, intra-molecular hydrogen bond formed between the carbonyl oxygen of the P2′ Phe and the amide nitrogen of the P5′ Met residues (Figure 5D), which is not present in the **I1**-bound complex. There are also hydrophobic interactions between the aromatic ring of the **JTH-NB72-39** P2′ Phe and BoNT/A LC residues previously found to form a hydrophobic pocket for binding by the larger, indol P2′ Trp moiety of **I1** [35] (Figure 6). For **JTH-NB72-39**, the BoNT/A LC side-chains of Leu 367 and Phe 369, together with the aliphatic portions of Asn 368, contribute to the formation of this hydrophobic pocket. Additionally, the aliphatic side-chain of Leu 256 interacts with the **JTH-NB72-39** P2′ Phe side-chain (Figure 6), but its electron density is weaker than observed in the BoNT/A LC:**I1** complex, suggesting that the larger **I1** P2′ Trp is better suited than the **JTH-NB72-39** P2′ Phe for stabilizing this residue and forming a non-polar binding site (Figure 6). This observation partially explains the lower potency of these three PLMs (Figure 1) relative to **I1**.

A new PLM component incorporated into the design of **JTH-NB72-39** also present in **JTH-NB72-35** and **JTH-NB72-38** is the *gem*-dimethyl-glycine residue, Aib, in the P3′ position (Figure 1). The rationale for incorporating this component was to stabilize the PLMs' observed 3_10_ helical conformation, as inferred from the **I1** binding mode [35], and to decrease the binding entropies of the new designs. In the present co-crystal structure, Aib engages in favorable *inter*molecular contacts (via its hydrophobic *gem*-dimethyl groups) with the side-chain of BoNT/A LC residue Val 70, and an *intra*molecular, stabilizing, hydrogen bond with the amide nitrogen of its P6′ Leu (not shown). In addition to these interactions, our structure reveals unexpected conformational restrains introduced by this Aib residue that affect neighboring residues in the PLM. The backbone atoms of the more potent **I1** PLM superimpose well with corresponding atoms found in **JTH-NB72-39** up to the P2′ position (Figure 6B). However, the backbone of **JTH-NB72-39** abruptly contracts forming a sharp bend at the P3′ position which results from the distinct conformational effects of the Aib residue. Based on this observation, we conclude that, in addition to possessing a smaller volume than the P2′ Trp group of **I1**, and the concurrent absence of an ionic indol group, the P2′ Phe of **JTH-NB72-39** is hindered in its orientation by the geometrical restraints imposed on the its backbone by the adjacent P3′ Aib residue.

The P4′ Ala residue of **JTH-NB72-39** engages in *intra*molecular, hydrophobic interactions with the side-chain methylenes of the PLM's P1 Arg and P6′ Met, as well as with the backbone amides of the PLM's P1′ Arg and P3′ Aib, while the P5′ Met engages in intermolecular interactions with BoNT/A LC residues Glu 257, Val 258, Ser 259, and Glu 262, and intra-molecular contacts with the P1 Arg, P2′ Phe, P4′ Ala, and P6′ Leu. The alkyl chain of the outermost residue in the inhibitor (i.e., **JTH-NB72-39** P6′ Leu), although solvent exposed, engages in favorable *intra*molecular interactions with the hydrophobic surfaces of its neighboring P2′ Phe and P3′ Aib PLM residues, as well as an inter-molecular interaction with the BoNT/A LC Phe 369 side-chain phenyl.

### BoNT/A LC substrate binding cleft plasticity

Silvaggi *et al.* observed structural plasticity in the BoNT/A LC substrate cleft upon binding to three different hydroxamate derivatives [48]. This plasticity has also been documented by the distinct binding contacts identified in subsequent complexes of the BoNT/A LC with other PLM inhibitors [39], [40]. Our co-crystal structure of **JTH-NB72-39** bound to the BoNT/A LC further underscores this plasticity. Importantly, the complexes of BoNT/A LC with **JTH-NB72-39** and with **I1** (PDB reference code 3DS9, [35]), and the unbound crystal structure (PDB reference code 3DSE [35]) crystallized in the same space group with very similar cell dimensions. Thus, we can make comparisons of conformations between these specific crystal structures without the potential influence of crystal packing contacts.

The BoNT/A LC's overall fold is similar in the bound and unbound forms: the **JTH-NB72-39**-bound form superimposes to the unbound form with a 0.58 Å r.m.s.d. for all Cα atoms. In the BoNT/A LC:**JTH-NB72-39** structure, the three LC residues that directly coordinate the Zn(II) ion in the active site, i.e., His 223, His 227 and Glu 262, maintain the same geometry and conformation observed in the unbound form of the LC protease. However, Glu 224 no longer associates with the Zn(II) ion via a ‘catalytic water’ molecule, as observed in all crystal structures of the unbound form of the enzyme; instead it interacts with the amino terminal nitrogen atom of the PLM via a H-bond (Figure 2C). Additional major differences between the bound and unbound structures are observed in the 20, 200, and 250 loops. Specifically, backbone atom differences observed for the 370 loop suggest **JTH-NB72-39**-induced conformational changes around residues Asn 368, Phe 369, and Asp 370, since similar arrangements of the symmetry mates near the 370 loop are observed for both bound (**JTH-NB72-39** and **I1**) and unbound forms of the LC. Residue Phe 369 moves closer to **JTH-NB72-39** than observed for any of the reported tetrameric peptides or RRATKM [39], [40]. This interaction is even more pronounced in the BoNT/A LC:**I1** co-crystal structure. Additionally, **I1** is also in closer proximity to BoNT/A LC residue Leu 256 [35] than **JTH-NB72-39** (Figure 6). These observations reinforce the hypothesis that the P2′ Trp residue in inhibitor **I1** is a more favorable ‘binding anchor’ than the corresponding P2′ Phe of **JTH-NB72-39**.

**Figure 7:**
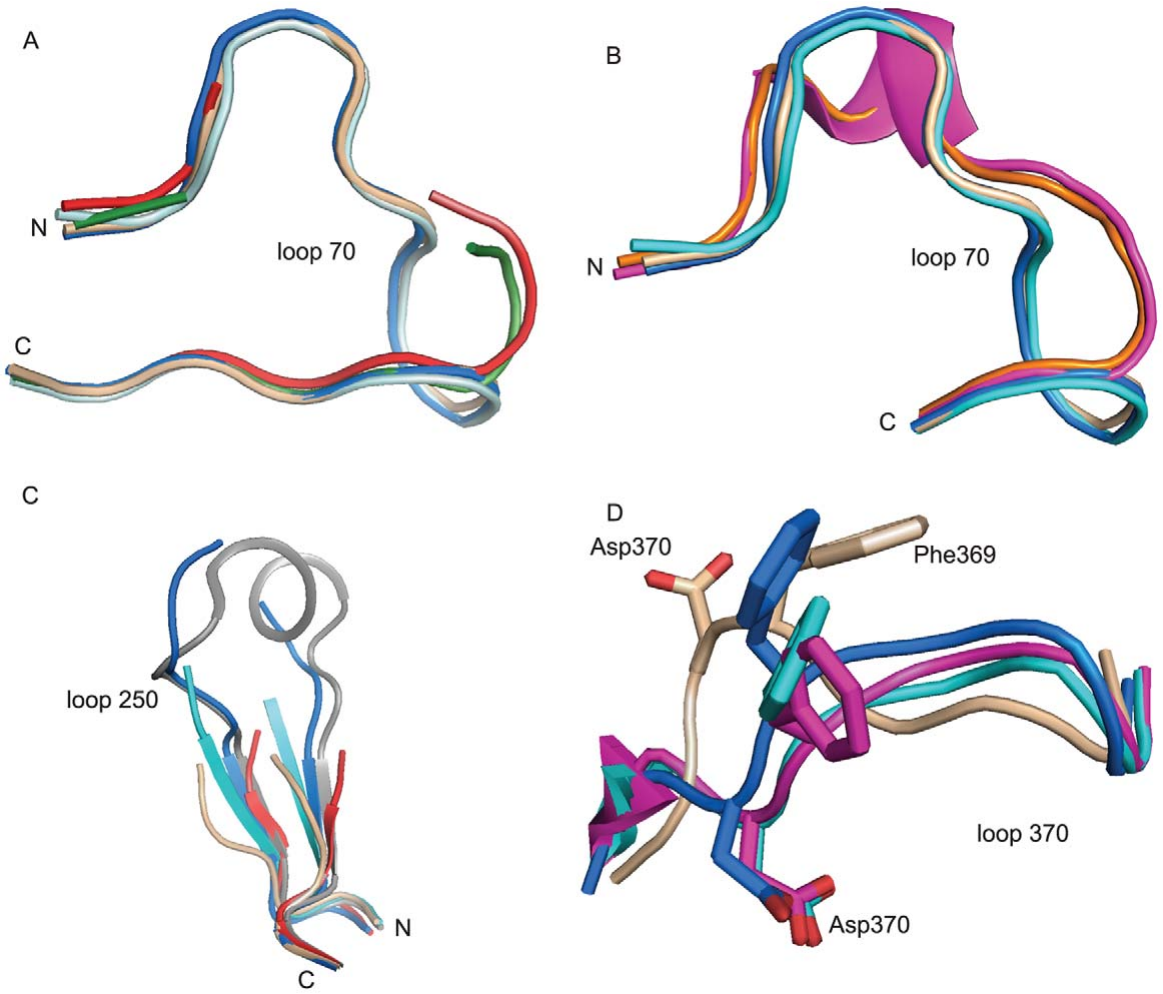
Observed plasticity in the BoNT/A LC substrate binding cleft. Superposition of: **A.** The 70 loop backbone of BoNT/A LC in the unbound (tan), **I1**- (blue), **JTH-NB72-39**- (cyan), ArgHX- (red), and CRATKML-bound forms (dark green). **B.** The 70 loop backbone of the BoNT/A LC in the unbound (tan), **I1**- (blue), **JTH-NB72-39**- (cyan), RRGM- (magenta), and RRATKM-bound (orange) forms. **C.** The 250 loop backbone of the BoNT/A LC in the unbound (tan), **I1**- (blue), **JTH-NB72-39**- (cyan), ArgHX- (red), and QRATKM- bound (grey) forms. **D.** The 370 loop backbone of the BoNT/A LC in the unbound (tan), **JTH-NB72-39**- (red), **I1**- (blue), and ArgHX-bound (cyan). The backbone is displayed in cartoon representation and the side chain of BoNT/A LC Phe 369 is shown as sticks.

In the unbound form of BoNT/A LC, residues 64–70 adopt a loop conformation by packing against the β strand formed by residues 415–420. This loop is unaltered in the **I1**- and **JTH-NB72-39**-bound structures (Figure 7A). However, for the crystal structures of the complexes with the hydroxamate derivatives and the CRATKML peptide [49], no electron density was observed for these residues (Figure 7A). Binding of the tetrapeptides, and of the QRATKM and RRATKM PLMs results in a significant displacement of the backbone in the 70 loop away from the active site (Figure 7B).

Electron density for the 250 loop of the BoNT/A LC is only observed for structures of the LC complexed with PLMs containing either no side chain (i.e. a Gly component) or an Ala in the P2′ position (Figure 7C). In contrast, no electron density is observed for various residues within this loop for either the unbound form of the BoNT/A LC, or when bound to arginine hydroxamate (ArgHX) (residues 245–256), **I1** (residues 250–253), or **JTH-NB72-39** (residues 247–255) (Figure 7C).

In all BoNT/A LC:inhibitor complexes described thus far, including the co-crystal described herein, there is a conformational change in the 367–372 residue segment of the 370 loop that is associated with rotamer changes in enzyme residues Phe 369 and Asp 370 (Figure 7D). The latter engages in the salt bridge (described above) with the Arg sidechain in the P1′ position of the inhibitors, a key specific interaction for binding of the SNAP-25 substrate and substrate-analog inhibitors. As for Phe 369, its side chain projects away from the binding cleft in the unbound form of the BoNT/A LC. By contrast, in the **JTH-NB72-39** complex, the center of this ring moves by ∼3.5 Å towards the binding cleft, facilitating the formation of a hydrophobic pocket that accommodates the Phe aromatic ring of the PLM. This conformational change for Phe 369 is even more pronounced in the BoNT/A LC:**I1** complex due to this PLM's larger P2′ Trp component (Figure 7D BoNT/A LC). In all other inhibitor complexes, Phe 369 adopts a conformation that differs when compared with either the unbound form of the enzyme, the **JTH-NB72-39**-, or the **I1**-bound complexes. Taken together, these conformational changes observed in the BoNT/A LC protease upon binding with different inhibitors reveal a highly ‘plastic’ binding cleft.

### Conformational helicity and PLM inhibitors of the BoNT/A LC

**Figure 8:**
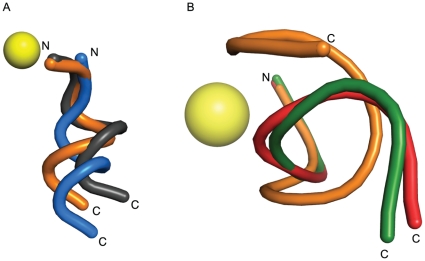
The helical chirality of PLM inhibitors. Superposition of the backbones (tube representation) of all BoNT/A LC PLM inhibitors reported to date (and which are longer than four residues). **A**. PLM inhibitors whose backbones display positive helical chirality upon binding to BoNT/A LC: **JTH-NB72-39** (orange), **I1** (grey), and CRATKML (blue). **B**. PLM inhibitors with negative helical chirality upon binding to BoNT/A LC: RRATKM (green), and QRATKM (red). **JTH-NB72-39** (orange) is shown for comparative purposes. The BoNT/A LC Zn(II) ion is shown as a yellow sphere.

Including the present structure, there are now five co-crystal structures of PLM-based inhibitors in complex with the BoNT/A LC that are longer than four residues. A common structural feature found in all five bound PLMs is a 3_10_ helical turn in the inhibitor's backbones (Table 2). As indicated above, **JTH-NB72-39** was designed to further stabilize this helical turn by introducing an Aib residue in the P3′ position. Indeed, there is a 3_10_ helical conformation for the backbone atoms of this PLM, although the P3′ Aib residue slightly distorts the helical turn and deviates from the canonical 3_10_-helix conformation adopted by **I1** (Figure 8A), likely by virtue of the unusual geometric constrains of this residue, i.e, the gem-dimethyl effect [50]. The electron density suggests that the helical pathway also includes the Met residue in the P5′ position, resembling the 3_10_ helix observed for residues P2′-P5′ in **I1**.

**Table 2:** Helicity in BoNT/A LC PLM inhibitors.

Inhibitor												
	JTH-NB72-39		I1		CRATKML		QRATKM		RRATKM		RRGX*	
Position	SecStr*	α *	SecStr	α	SecStr	α	SecStr	α	SecStr	α	SecStr	α
P1	L		L		L		L		L		L	
P1′	L		L		L		L	−	L	−	L	−
P2′	H	+	H	+	H	+	L	−	L	−	L	
P3′	H	+	H	+	H	+	S	−	L	−	L	
P4′	H	+	H	+	H	+	L		L			
P5′	H		H		H		L		L			
P6′	L		L		L							

*Abbreviations:

SecStr: observed secondary structure from pdb coordinates (L: loop; H: Helix; S: Bend).

α: helical chirality (dihedral α angle – positive angle corresponds to a right-handed helix).

RRGX: any of the tetrapeptides reported in [51].

All SS and α values were calculated with the DSSPcont program DSSPcont: Continuous secondary structure assignments for proteins [61].

A helical backbone conformation is also observed in the Zn-chelating CRATKML PLM[49], similarly encompassing positions P2′ throughout P5′ (Figure 8A). Remarkably, only PLMs containing a Leu in the P6′ position adopt a right-handed 3_10_ α-helical conformation, in contrast to two closely related hexapeptide PLMs lacking a terminal Leu residue (Table 2) [39]. Instead, the φ and ψ torsion angles for QRATKM and RRATKM do not correspond to a canonical right-handed helical conformation (Figure 8B and Table 2). The backbone atoms of both peptides undergo a geometrical ‘bend’ which is most conspicuous along the backbone of the Thr residue in QRATKM (Table 2 and Figure 8B). Whereas inhibitors CRATKML, **I1** and **JTH-NB72-39** display a common right handedness (or positive chirality) of canonical α-helices, the QRATKM and RRATKM PLMs display unusual negative values in their dihedral α angles (i.e. negative helical chirality) (Table 2). The helical segments observed for inhibitors **I1**, **JTH-NB72-39**, and CRATKML are of similar length and entail the same residue positions (i.e. P2′-P5′); nevertheless, the CRATKML “helix” is slightly shifted relative to the other two PLMs due to Zn-coordination by its thiol group in the P1 Cys. Interestingly, PLMs QRATKM and RRATKM, which do not adopt a canonical helical conformation, are less potent (K_i_ values  = 133 µM and 95 µM, respectively) than the ‘helical’ PLMs **JTH-NB72-39**, **I1**, and CRATKML all of which possess nM range K_i_ values (Table S1). Hence, it is likely that the P6′ Leu residue in the helical PLMs not only stabilizes their helical conformations, but also increases inhibitory potency via increased occupation of the enzyme's substrate binding cleft. Together with the favorable hydrophobic interactions of the substituents at the P2′ position of **JTH-NB72-35**, **-38**, **-39**, and **I1**, the presence of a seventh residue might account for the higher potency of these PLMs over the hexa- or tetrapeptides reported by other groups (Table S1) [39], [51].

### Inhibition mechanism of JTH-NB72-39

The PLM **JTH-NB72-39** shares the same inter-molecular contacts that facilitate the mechanism of inhibition with other PLMs (Figure 5B), in particular **I1**, as previously described [35]. Specifically, all PLMs possessing a free amino terminus orient this substituent such that it engages in contacts with the proton shuttle Glu 224 side-chain carboxylate (Figure 5A–C). In this way, Glu 224 is no longer capable of ionizing the ‘catalytic’ water molecule and, as a consequence, it is hampered from using the protons from this water to catalyze the final cleavage of the scissile bond. In addition, all reported PLMs exhibit simultaneous, substrate-like interactions between their P1 residue backbone carbonyl oxygens and both, the side-chain hydroxyl group of Tyr 366 and the Zn(II) ion (Figure 5A–C). Furthermore, BoNT/A LC residue Arg 363 engages in electrostatic contacts with the peptidic backbones of reported PLMs (Figure 5A). The RRGL, RRGI, RRGM, RRGC, and RRATKM peptides [39], [40] consistently contact Arg 363 via their P1′ carbonyl oxygen (Figure 5A). **JTH-NB72-39**, on the other hand, forms this interaction indirectly, via a bridging water molecule (Figure 5A). This difference is most likely due to the geometric restraints imposed by the aromatic group in the P2′ position of **JTH-NB72-39** (which is either a Gly or an Ala in all other PLM inhibitors [39], [40]).

The CRATKML inhibitor conformation deviates from those of all the other PLMs. It is likely that the Zn-coordinating geometry of the P1 Cys of CRATKML shifts the other interactions in the complex (Figure 5C) [40]. For its first residue, P1 Cys, the terminal amino group of this PLM is not proximal to the enzyme's proton shuttle - Glu 224, but rather, this P1 Cys engages in contacts with BoNT/A LC residue Arg 363 through its P1 carbonyl oxygen, as opposed to the P1′ carbonyl oxygen as observed for other PLMs [39], [40] (Figure 5A and 5C). As a result, CRATKML also lacks the hydrogen bond with Tyr 366 that is detected in all other PLM co-crystal structures (Figure 5C).

### Hypotheses for improving PLM inhibitory efficacies

The PLMs described here were designed in an attempt to improve potency displayed by previously reported PLM **I1** via the incorporation of a P1 position Arg residue (to engage in direct electrostatic interactions with BoNT/A LC acidic residues), and the incorporation of an Aib residue (to stabilize the inhibitors helical conformations) [35]. However, neither **JTH-NB72-35** (K_i_ = 315.5±28.6 nM), **JTH-NB72-38** (K_i_ = 990.5±116.9 nM), nor **JTH-NB72-39** (K_i_ = 638±92.0 nM) (Figure 1) are as potent as **I1** (K_i_ = 41 nM) [35]. The co-crystal structures of **JTH-NB72-39** and **I1** in complex with BoNT/A LC explain this surprising result. The higher K_i_ value for **JTH-NB72-39** compared to **I1** is, in part, due to fewer favorable hydrophobic contacts provided by the PLM's Phe component in the P2′ position versus the larger P2′ Trp of **I1**. Specifically, the **I1** Trp component is more efficient in coalescing the non-polar side-chains of BoNT/A LC residues Leu 256 and Phe 369 in their common binding site (Figure 6). Additionally, while the aliphatic side-chain of Leu 256 does contact the **JTH-NB72-39** P2′ Phe, the electron density for the side-chain of this BoNT/A LC residue is weaker than that observed in the BoNT/A LC:**I1** complex [35]. Hence, the **I1** P2′ Trp residue is more efficient for inducing the formation of this non-polar pocket than the P2′ Phe residue of JTH-NB72-39. Another unexpected result from our co-crystal structure is that the Aib residue introduces a slight deformation of the canonical 3_10_ helical backbone conformation observed for **I1** (Figure 7A).

**Figure 9:**
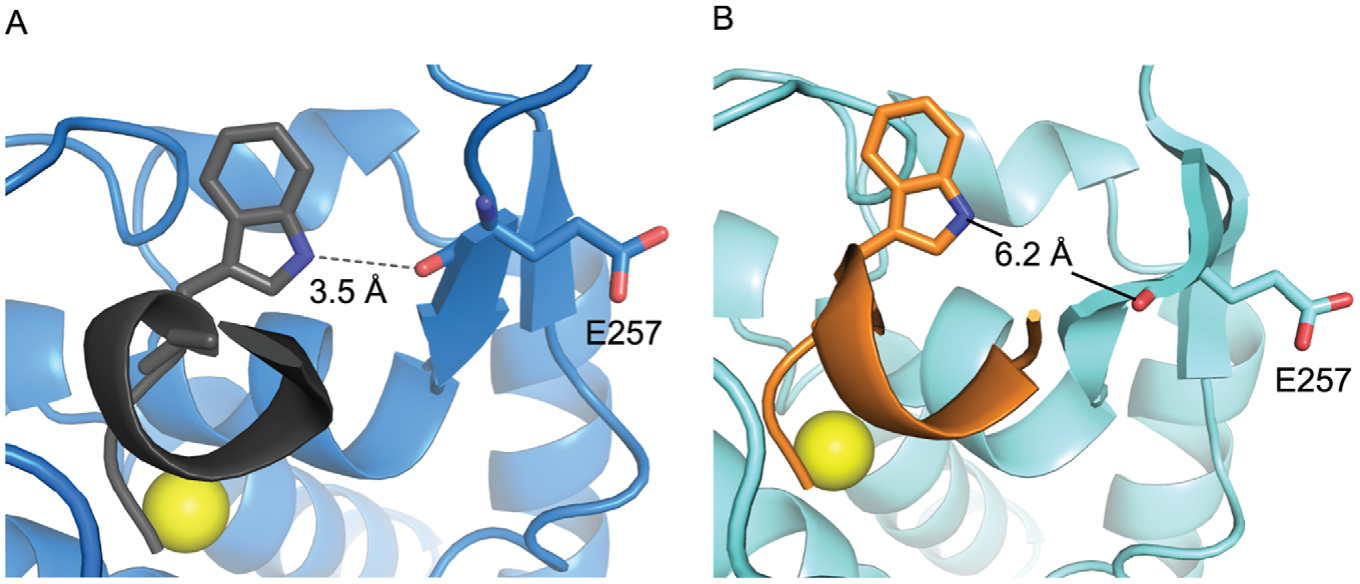
Modeling of a Trp residue in the P2′ position of JTH-NB72-39. **A.** Cartoon representation of the BoNT/A LC:**I1** complex. The BoNT/A LC is shown in blue, and **I1** in dark grey. The dashed line represents an H-bond between the indole nitrogen atom of the P2′ Trp and the carbonyl group of BoNT/A LC residue Glu 257. The H-bond distance is indicated. **B.** Cartoon representation of the BoNT/A LC:**JTH-NB72-39** complex. A Trp side chain has been modeled in the P2′ position (instead of the actual Phe side chain of **JTH-NB72-39**). The Trp rotamer model shown here positions the indole nitrogen as can bind in the closest possible proximity to the carbonyl group in Glu 257 (employing the BoNT/A LC: **JTH NB72-39** co-crystal). The solid line indicates the distance (not contact) between these two groups. The BoNT/A LC is shown in cyan, and **JTH-NB72-39** carbons and backbone ribbon in orange. The side chains of the P2′ Trp in the PLMs and Glu 257 in BoNT/A LC are displayed in stick representation in both panels. The yellow spheres represent the Zn(II) ion in both panels.

Our co-crystal structure also explains the different potencies observed for the other two PLMs (Figure 1). In particular, the most potent of the three reported PLMs (i.e., **JTH-NB72-35**) possesses a P2′ naphthalene methylene substituent, which would more efficiently bring together, and engage in more favorable hydrophobic contacts with, the non-polar side-chains of BoNT/A LC residues Leu 256 and Phe 369 (versus **JTH-NB72-39**), as well as with the aliphatic portions of the side-chain of residue Asn 368 at the enzyme binding site. Finally, it is likely that the same hypothesis applies to **JTH-NB72-38**; however, while the **JTH-NB72-38** P2′ Trp indole does provide a larger ring system for binding in the indicated BoNT/A LC hydrophobic pocket (see above), the polar pyrrole nitrogen atom is unable to engage in a favorable hydrogen bond with the backbone carbonyl of BoNT/A LC residue Glu 257, an interaction that was previously observed in the BoNT/A LC:**I1** complex (Figure 9A) [35]. Instead, modeling of a Trp side chain in the P2′ position of the **JTH-NB72-39** PLM revealed that none of the rotamers available for the - CH_2_-indole side chain positions it so that it engages in a hydrogen bond with any residue in the enzyme (Figure 9B). Indeed, our analyses indicated that the polar nature of the **JTH-NB72-38** pyrrole nitrogen results in unfavorable hydrophobic-polar clashes in the hydrophobic pocket indicated above. Hence, this analysis provides a rational basis for explaining the higher K_i_ compared of this PLM versus those of **JTH-NB72-35**, **JTH-NB72-39**, and **I1**.

The decreased inhibitory efficacies of all three JTH PLMs compared to **Il** also appears to be partially due to the fact that they all incorporate an Arg residue at the P1 versus the P1 – P2′ DNP-DAP component of **I1**. In particular, the rigidity of the DNP phenyl and the solvation of its two nitro functional groups may be necessary for stabilizing this PLM's helical structure upon binding in the enzyme's substrate cleft. This, in turn, would decrease **I1**′s binding entropy. By comparison, the flexible side-chain of the P1 Arg of the JTH PLMs does not provide the same helix-stabilizing character. Additionally, the JTH PLMs lack the DAB component found in **I1**. In the BoNT/A LC:**I1** co-crystal structure, this cationic component engages in a hydrogen bond with the side-chain amide of BoNT/A LC residue Gln 162 [35]. Comparatively, in the BoNT/A LC:**JTH-NB72-39** co-crystal structure, the PLM's non-polar P4′ Ala residue cannot engage in such a favorable H-bond. Finally, the initial weak electron density observed for the C-terminal residues of the JTH PLMs indicates that these residues may be destabilizing the overall binding modes of these inhibitors via entropic contributions, and therefore, other components that engage in more definitive hydrophobic and/or polar contacts with the enzyme cleft would be necessary for improving future PLM potencies.

Based on available structural and mechanistic data, future PLM designs will focus on optimizing the P1 - P2′positions, while simultaneously introducing changes to terminal PLM residues/components to decrease their entropic contributions. Moreover, future designs will also incorporate peptidomimetic features that will increase the drug-like character of the PLMs.

### Conclusion

The design and synthesis of three new PLM inhibitors, which are pivotal for guiding the development of peptidomimetics and SMNPIs, have been presented. In order to characterize the binding modes for the PLMs, a co-crystal structure of one, **JTH-NB72-39**, was determined, which possesses components that have been independently reported to directly interact with the active site of the BoNT/A LC [35], [39], [40]. Based on comparisons between the binding mode determined for **JTH-NB72-39** with other non-Zn-chelating BoNT/A LC:PLM co-crystal structures [35], [39], [40], a consistent inhibition mechanism has emerged [35]. Specifically, we provide a unifying PLM-based mechanism of action: in all cases the presence of a P1 amino terminal residue is key for effectively ‘arresting’ the proteolytic activity of the BoNT/A LC. This discovery explains why SNAP-25 substrates N-terminally extended beyond the P1 position are cleaved by the BoNT/A LC[46]; in such peptides, due to their participation in a peptide bond with the P2 residue, the P1 amino group becomes an amide, rendering it non-competent for ‘locking’ the Glu 224 carboxylate group [39]. This observation emphasizes the requirement of a highly ionizable group in this position in order to strongly interact with Glu 224 – to effectively inhibit the BoNT/A LC protease and will be important for guiding the rational design of new PLMs and peptidomimetics, as well as for the discovery of new SMNPIs and the synthetic optimizations of existing SMNPIs.

The **JTH-NB72-39**-BoNT/A LC co-crystal structure presented here, and its comparison with all other SNAP-25-derived PLM inhibitor co-crystal structures known to date, highlights the importance of the BoNT/A LC 370 loop for substrate binding and cleavage specificity. It is this loop that contains the Asp 370 residue, which is pivotal for substrate discrimination. Also, BoNT/A LC residue Phe 369 is located in this region, and according to the structure presented here and the co-crystal structure with **I1** [35], forms part of a hydrophobic pocket that efficiently anchors non-polar groups located in the P2′ position of these PLMs. Future designs will involve PLM components that can further stabilize a helical backbone orientation without interfering with binding, as well as the incorporation of bulkier non-polar components at the P2′ position.

## Methods

### Synthesis

#### General

*N,N*-Diisopropylethylamine was sequentially distilled from ninhydrin then KOH and stored under argon. Piperidine was distilled from CaH_2_ and stored under argon. Phenol was purified by dissolving the solid in diethyl ether, washing with a saturated aqueous solution of NaHCO_3_ (3x), extracting with aqueous NaOH (0.1 M) (3x), acidifying the aqueous extracts with 0.1 N HCl, extracting with Et_2_O (3x), concentrating the ethereal extracts under reduced pressure, and the dry solid was stored under argon. *N,N-*Dimethylformamide was purchased from Alfa Aesar as anhydrous and amine free in 4 L quantities and stored in 1 L Amber bottles (dried overnight in an oven at 140°C) over activated 4 Å molecular sieves under argon. Trifluoroacetic acid (biochemical grade, 99.5+% pure) was purchased from Alfa Aesar and used as received. Methanol (HPLC grade), water (HPLC grade), and thioanisole (99% purity) were purchased from Aldrich and used as received. Triisopropylsilane (99% purity) was purchased from Acros and used as received. 1,2-ethanedithiol (>98% pure) was purchased from Fluka and used as received. All natural Fmoc-protected amino acids and 3-(diethoxy-phosphoryloxy)-3*H*-benzo[d][1], [2], [3]-triazin-4-one were purchased from either Peptides International or Advanced Automated Peptide Protein Technologies (AAPPTEC) and used as received. Unnatural Fmoc protected amino acids and Rink Amide Resin SS, 100–200 mesh, 1% DVB were purchased from Advanced Chemtech and used as received. BD Falcon BlueMax 50 mL graduated tubes and 25 mm syringe filters with a 0.45 µm nylon frit were purchased from Fischer Scientific.

The Fmoc-solid phase peptide syntheses were performed on a CEM Discover manual microwave peptide synthesizer fitted with a fiber-optic temperature probe. Solid phase peptide syntheses were performed in a 25 mL polypropylene reaction vessel. The 25 mL polypropylene reaction vessel was constructed by inserting a Teflon ring (0.4 mm height, 2.1 mm outer diameter, 1.8 mm inner diameter) into a capped 25 mL SPE reservoir purchased from Grace Davison Discovery Science (Catalogue #: 210425) containing a frit purchased from Grace Davison Discovery Science (Catalogue #: 211416) (Figure S1).

Preparative reverse phase HPLC purifications were performed on a Gilson HPLC system with 220 and 254 nm UV detection, using a Phenomenex Luna 5µ C18(2) 100 Å, AX (75×30.0 mm) column at a flow rate of 10 mL/min. Unless otherwise noted, all preparative runs used linear gradients of 30–60% buffer B in A (A: water containing 0.1% TFA, B: CH_3_CN containing 0.1% TFA) over 30 min. Analytical HPLC traces of final products were performed on a Gilson HPLC system with 220 and 254 nm UV detection, using a Varian Microsorb 100-3 C18 (100×4.6 mm) column at a flow rate of 0.7 mL/min. Unless otherwise noted, all analytical runs used linear gradients of 30–100% buffer B in A (A: water containing 0.1% TFA, B: MeOH) over 70 min. CD Spectra were recorded on a Jasco J-815 Circular Dichroism Spectrometer. Unless otherwise noted, all CD spectra were recorded in MeOH at a concentration of 0.5 mM, at 298 K, over a range of 300–200 nm, at a scan rate of 50 nm/min. Mass spectra were obtained using MALDI TOF/TOF with 2,5-dihydroxybenzoic acid as the matrix in the positive ion mode. Lyophilization was accomplished using a Labconco FreeZone 4.5 liter bench top freeze dry system. Centrifugation was accomplished using a Sorvall RT-7 Plus bench top centrifuge.

Proton and carbon NMR spectra were recorded using a Bruker Avance spectrometer at 600 MHz/150 MHz (^1^H NMR/^13^C NMR) in D_2_O (298 K), unless otherwise noted. Chemical shifts (δ) are reported in parts per million (ppm) using MeOH solvent peaks as an internal reference (referenced to 3.34 ppm (^1^H) and 49.5 ppm (^13^C)). ^1^H NMR data are reported as follows: chemical shift, multiplicity (s = singlet, d = doublet, t = triplet, q = quartet, m = multiplet, dd = doublet of doublets, dt = doublet of triplets, td = triplet of doublets, qd = quartet of doublets), coupling constants (*J*) in Hertz (Hz), and integration. ^13^C NMR spectra were obtained using a proton-decoupled pulse sequence with d1 of 6 sec, and are tabulated by observed peak.

A stock solution of the coupling base was prepared by dissolving DIPEA (1.74 mL, 1.00 mmol) in DMF (5.00 mL) to give a 0.148 M solution. A stock solution of the Fmoc-cleavage base was prepared by dissolving piperidine (1.00 mL, 10.1 mmol) in DMF (4.00 mL) to give a 2.02 M solution. A stock solution of the resin cleavage cocktail was prepared by combining TFA (5.07 g, 44.5 mmol), PhSCH_3_ (0.210 g, 1.69 mmol), PhOH (0.215 g, 2.28 mmol), TIPSH (0.0620 g, 0.392 mmol), 1,2-EDT (0.135 g, 1.43 mmol) and H_2_O (0.100 g, 5.56 mmol). All stock solutions were freshly prepared prior to use.

#### General procedure A: solid phase peptide synthesis

To a 25 mL polypropylene reaction vessel charged with a Teflon stir bar (10×3 mm) was added the Rink Amide resin (0.143 g, 0.100 mmol, loading 0.700 mmol/g, 1.00 equiv). The resin was washed with MeOH (2×5 mL), CH_2_Cl_2_ (3×10 mL) and DMF (3×10 mL), suspended in CH_2_Cl_2_ (5 mL) and allowed to swell at room temperature for 30 min. The resin was filtered and washed with DMF (3×10 mL). The Fmoc group was cleaved by heating the resin in the Fmoc-cleavage base stock solution (1 mL) in the microwave (35 W, 78°C, 3 min). The resin was filtered and washed with DMF (3×10 mL), CH_2_Cl_2_ (3×10 mL) and DMF (3×10 mL). The first Fmoc protected amino acid was coupled to the resin by heating in a pre-mixed solution of amino acid (0.350 mmol, 3.50 equiv), DEPBT (0.105 g, 0.350 mmol, 3.50 equiv), DMF (0.80 mL), and Fmoc-coupling base stock solution (0.75 mL) in the microwave (25 W, 80°C, 5 min). The resin was filtered and washed with DMF (3×10 mL), CH_2_Cl_2_ (3×10 mL) and DMF (3×10 mL). The Fmoc group was cleaved as previously described, and the next amino acid was coupled. This process of Fmoc cleavage and amino acid coupling was repeated for each additional amino acid. After the final Fmoc cleavage, the resin was washed with DMF (30 mL) and CH_2_Cl_2_ (20 mL). The protecting groups were cleaved by treatment of the dry resin with the resin cleavage cocktail stock solution (2.50 mL) for 2 h at room temperature with vigorous stirring. The resin was filtered and rinsed with the remaining resin cleavage cocktail stock solution (1.50 mL) and TFA (1.50 mL), collecting the filtrate and rinses in a 50 mL BD Falcon tube. The sample was concentrated to a heterogeneous mixture (approximately 0.2 mL) under a stream of argon for 30 min. Cold diethyl ether (45 mL) was added to precipitate the crude peptide. The sample was centrifuged (3200 rpm, −8°C, 15 min) and the supernatant was discarded. The crude peptide was transferred to a 20 mL scintillation vial with approximately 5 mL of a mixture of H_2_O/CH_3_CN (9∶1) and lyophilized overnight. The crude peptide was dissolved in H_2_O containing 0.1% TFA (5.00 mL) and filtered through a 0.45 µm nylon syringe filter. The filtrate was purified by preparative RP HPLC.

#### JTH-NB72-35 synthesis

Prepared according to general procedure A utilizing the following amino acid sequence: Fmoc-L-Leu-OH (0.124 g, 0.350 mmol, 3.50 equiv), Fmoc-L-Met-OH (0.130 g, 0.350 mmol, 3.50 equiv), Fmoc-L-Ala-OH (0.115 g, 0.350 mmol, 3.50 equiv), Fmoc-Aib-OH (0.114 g, 0.350 mmol, 3.50 equiv), Fmoc-L-1-Nal-OH (0.153 g, 0.350 mmol, 3.50 equiv), Fmoc-L-Arg(Pbf)-OH (0.227 g, 0.350 mmol, 3.50 equiv), Fmoc-L-Arg(Pbf)-OH (0.227 g, 0.350 mmol, 3.50 equiv). **JTH-NB72-35** (0.0399 g, 40%) was obtained as a white powder: The product was characterized by ^1^H NMR (Table S2); ^13^C NMR (Table S2); DEPT-135; COSY; HMBC; HMQC; HPLC RT 5.7 min, HRMS (MALDI^+^) *m/z* calcd for C_43_H_71_N_14_O_7_S [M+H] 927.5351, Found 927.5355. Figures S2 and S3 provide the CD spectrum and HPLC trace, respectively, for this PLM.

#### JTH-NB72-38 synthesis

Prepared according to general procedure A utilizing the following amino acid sequence: Fmoc-L-Leu-OH (0.124 g, 0.350 mmol, 3.50 equiv), Fmoc-L-Met-OH (0.130 g, 0.350 mmol, 3.50 equiv), Fmoc-L-Ala-OH (0.115 g, 0.350 mmol, 3.50 equiv), Fmoc-Aib-OH (0.114 g, 0.350 mmol, 3.50 equiv), Fmoc-L-Trp(Boc)-OH (0.185 g, 0.350 mmol, 3.50 equiv), Fmoc-L-Arg(Pbf)-OH (0.227 g, 0.350 mmol, 3.50 equiv), Fmoc-L-Arg(Pbf)-OH (0.227 g, 0.350 mmol, 3.50 equiv). **JTH-NB72-38** (0.0415 g, 41%) was obtained as a white powder: The product was characterized by ^1^H NMR (Table S3); ^13^C NMR (Table S3); DEPT-135; COSY; HMBC; HMQC; HPLC RT 5.7 min, HRMS (MALDI^+^) *m/z* calcd for C_41_H_70_N_15_O_7_S [M+H] 916.5303, Found 916.5461. Figures S4 and S5 provide the CD spectrum and HPLC trace, respectively, for this PLM.

#### JTH-NB72-39 synthesis

Prepared according to general procedure A utilizing the following amino acid sequence: Fmoc-L-Leu-OH (0.124 g, 0.350 mmol, 3.50 equiv), Fmoc-L-Met-OH (0.130 g, 0.350 mmol, 3.50 equiv), Fmoc-L-Ala-OH (0.115 g, 0.350 mmol, 3.50 equiv), Fmoc-Aib-OH (0.114 g, 0.350 mmol, 3.50 equiv), Fmoc-L-Phe-OH (0.136 g, 0.350 mmol, 3.50 equiv), Fmoc-L-Arg(Pbf)-OH (0.227 g, 0.350 mmol, 3.50 equiv), Fmoc-L-Arg(Pbf)-OH (0.227 g, 0.350 mmol, 3.50 equiv). **JTH-NB72-39** (0.0318 g, 34%) was obtained as a white powder: The product was characterized by ^1^H NMR (Table S4); ^13^C NMR (Table S4); DEPT-135; COSY; HMBC; HMQC; HPLC RT 5.6 min, HRMS (MALDI^+^) *m/z* calcd for C_39_H_68_N_14_NaO_7_S [M+Na] 899.5014, Found 899.5021. Figures S6 and S7 provide the CD spectrum and HPLC trace, respectively, for this PLM.

### *In vitro* testing

The HPLC-based assay used to calculate PLM inhibition constants has been published extensively [46], [52], [53], [54], [55], [56]. In brief, the assay utilizes an N-terminal acetylated, C-terminal aminated, synthetic peptide identical in sequence to residues 187–203 of SNAP-25. Substrate hydrolysis is determined by HPLC separation of the products from the substrate, followed by measurement of the peak areas. Assay mixtures consisted of 40 mM HEPES–0.05% Tween (pH 7.3), recombinant BoNT/A LC, peptide substrate, 0.5 mg/ml Bovine Serum Albumin, and various PLM concentrations. Assays were run at 37°C, quenched by the addition of TFA, and analyzed by reverse-phase HPLC. To eliminate Zn chelating agents, the assay is run in the presence of excess Zn (50 µM). K_i_ values were calculated by measuring PLM mediated inhibition at different substrate concentrations and treating the kinetic data by the method of Dixon. Inhibition constants (i.e. K_i_ values) were extracted from the slopes of Dixon plots: K_i_  =  K_m_/(slope x V_max_ x S), where S is the substrate concentration. All reported values are averages of at least three independent determinations using nine PLM concentrations.

### X-ray crystallography and structural analysis

#### BoNT/A LC: PLMs mixture preparation

Details of the bacterial expression and purification of the active form of wt BoNT/A LC used in this study have been previously described[45]. Stock solutions of wt BoNTA-LC containing 20 mM HEPES, pH 7.4 were adjusted to a final 150 µM protein concentration. Lyophilized **JTH-NB72-35, -38, and -39** inhibitors were resuspended in distilled water to a final 10 mM concentration. Individual mixtures of BoNT/A LC and each of the three PLMs were prepared by mixing both stock solutions to attain a final 50 µM BoNT/A LC and 1 mM PLM concentrations.

#### Crystallization and data collection

Crystals were obtained by using the hanging drop vapor diffusion method at 20°C. Briefly, 3 µL of a mixture of 50 µM BoNT/A and 1 mM of each PLM inhibitor were mixed with 1.5 µL of the mother liquor containing 14% PEG MME 2000, 10 mM NiCl_2_, and 100 mM HEPES pH 8.5. A layer of a 1∶1 mixture of paraffin:silicon oil was overlaid onto the mother liquor present in the well. Crystals for the BoNT/A LC:**JTH-NB72-39** mixture appeared after approximately five days of incubation and they were directly transferred into a cryo-solution containing 25%(v/v) PEG600, 0.14 X PEG MME 2000, 10 mM NiCl_2_, and 100 mM HEPES pH 8.5, and then flash-frozen in liquid nitrogen. Only microcrystals were observed for the other two PLMs, and further manipulation did not result in any improvement of their size. The diffraction data were collected at beamline 11.1 of the SSRL (Stanford Synchrotron Radiation Laboratory) at a wavelength of 1 Å, and at a temperature of 100 °K. The diffracted crystals belonged to the P2_1_2_1_2 group. Integration, indexing, and scaling of the diffraction data was performed using the HKL2000 suite of programs [57].

#### Structure determination and refinement of the wt BoNT/A LC:JTH-NB72-39 complex

The coordinates in the 1XTF pdb file were used as the search model to determine the structure of the wt BoNT/A LC:**JTH-NB72-39** complex by molecular replacement using the PHASER module in CCP4i [58]. The initial values for the R_work_ and R_free_ of the generated model were 27.1% and 31.3%, respectively. The σ_A_-weighted mF_o_-F_c_ electron density map clearly indicated the presence of **JTH-NB72-39** in the vicinity of the active site (Fig. 2). The coordinates of the **JTH-NB72-39** inhibitor were then added to those of the BoNT/A LC in the structure of the complex using Coot[59]. Final refinement and modeling was performed using Phenix [60]. The quality of the final structure was assessed using MolProbity. Ramachandran analysis showed that the BoNT/A LC:**JTH-NB72-39** structure had 97.14% residues in the favored region with no outliers. The coordinates and structure factors have been deposited in the PDB (ID 3NF3).

### Analysis of the secondary structure of PLM inhibitors

In order to determine the secondary structure of all the PLM inhibitors reported to date, the pdb files for their complexes with BoNT/A LC were used as input for the DSSPcont program [61]. The output of this analysis was used to build Table 2.

## Supporting Information

Figure S1 Diagram of assembled 25 mL polypropylene reaction vessel.
(0.11 MB DOC)

Figure S2 CD spectrum of JTH-NB72-35 (0.5 mmol) in MeOH.
(0.38 MB DOC)

Figure S3 Analytical HPLC trace of JTH-NB72-35 using a linear gradient of 30–100% buffer B in A (A: water containing 0.1% TFA, B: MeOH) over 70 min with UV detection at 220 nm.
(0.14 MB DOC)

Figure S4 CD spectrum of JTH-NB72-38 (0.5 mmol) in MeOH.
(0.37 MB DOC)

Figure S5 Analytical HPLC trace of JTH-NB72-38 using a linear gradient of 30–100% buffer B in A (A: water containing 0.1% TFA, B: MeOH) over 70 min with UV detection at 220 nm at a flow rate of 0.7 mL/min.
(0.14 MB DOC)

Figure S6 CD spectrum of JTH-NB72-39 (0.5 mmol) in MeOH.
(0.37 MB DOC)

Figure S7 Analytical HPLC trace of JTH-NB72-39 using a linear gradient of 30–100% buffer B in A (A: water containing 0.1% TFA, B: MeOH) over 70 min with UV detection at 220 nm at a flow rate of 0.7 mL/min.
(0.14 MB DOC)

Table S1 Potencies of structurally characterized BoNT/A LC inhibitors.
(0.07 MB DOC)

Table S2 1H and 13C NMR Data for JTH-NB72-35 (Figure 1) (600 MHz/150 MHz) in D2O (298 K) with MeOH as an internal reference (referenced to 3.34 ppm (1H) and 49.5 ppm (13C)).
(0.13 MB DOC)

Table S3 1H and 13C NMR Data for JTH-NB72-38 (Figure1) (600 MHz/150 MHz) in D2O (298 K) with MeOH as an internal reference (referenced to 3.34 ppm (1H) and 49.5 ppm (13C)).
(0.14 MB DOC)

Table S4 1H and 13C NMR Data for JTH-NB72-39 (Figure1) (600 MHz/150 MHz) in D2O (298 K) with MeOH as an internal reference (referenced to 3.34 ppm (1H) and 49.5 ppm (13C)).
(0.08 MB DOC)
