# Supplementary figures and images for: Iterative Structure-Based Peptide-Like Inhibitor Design against the Botulinum Neurotoxin Serotype A
Source: PLoS One. 2010 Jun 30;5(6):e11378. doi: 10.1371/journal.pone.0011378 (PMC2894858; doi:10.1371/journal.pone.0011378)

Figure S1


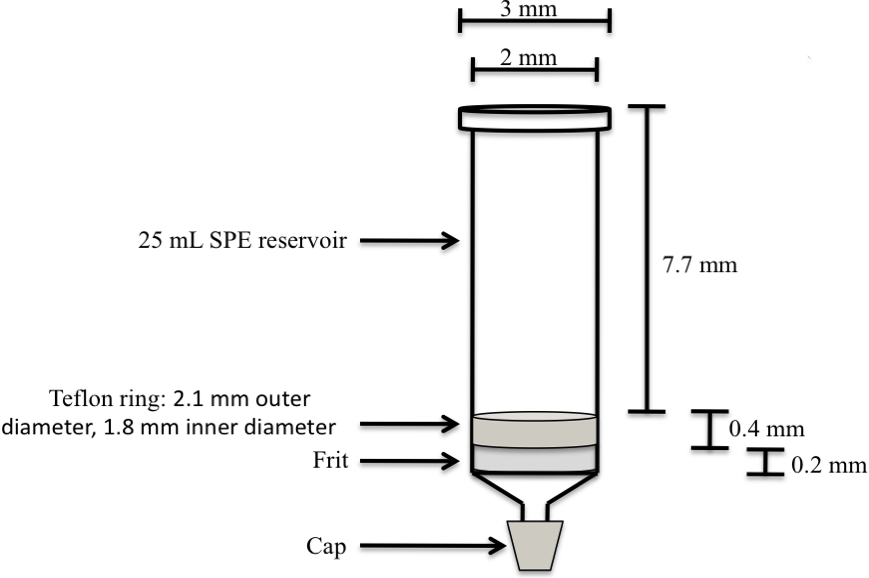

Supplement: Figure S1 — Diagram of assembled 25 mL polypropylene reaction vessel. (0.11 MB DOC) [file pone.0011378.s001.doc]

Figure S2


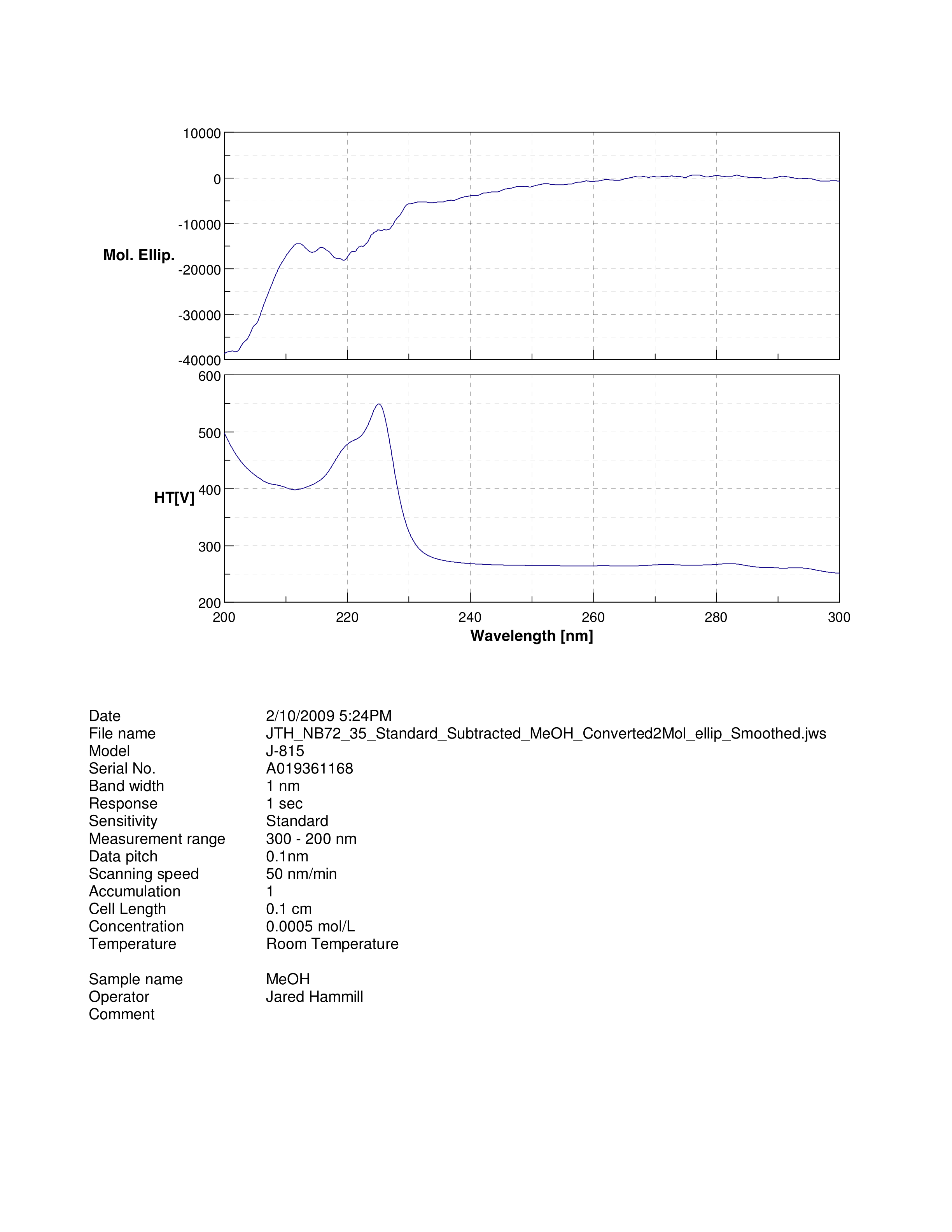

Supplement: Figure S2 — CD spectrum of JTH-NB72-35 (0.5 mmol) in MeOH. (0.38 MB DOC) [file pone.0011378.s002.doc]

Figure S3


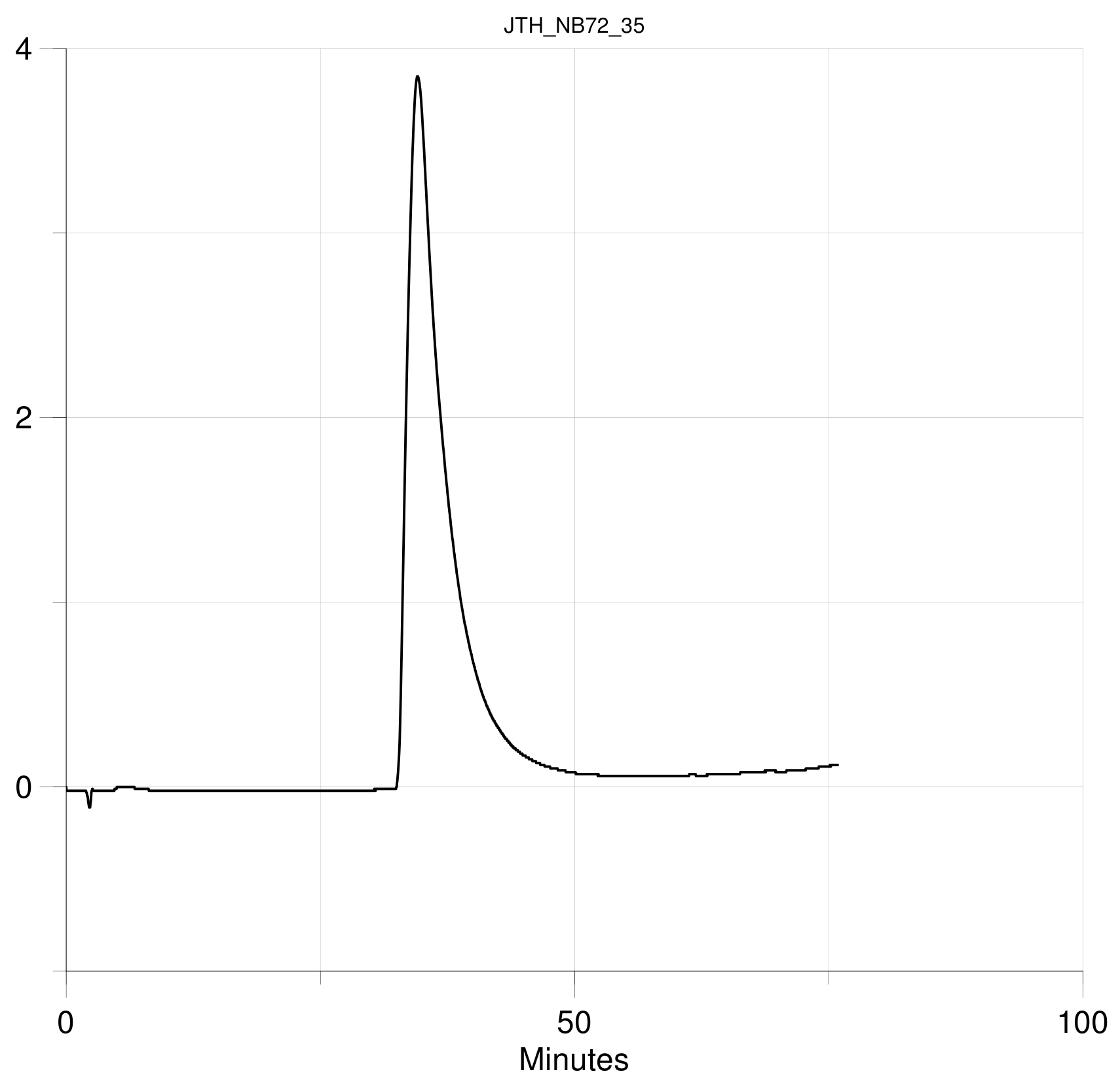

Supplement: Figure S3 — Analytical HPLC trace of JTH-NB72-35 using a linear gradient of 30–100% buffer B in A (A: water containing 0.1% TFA, B: MeOH) over 70 min with UV detection at 220 nm. (0.14 MB DOC) [file pone.0011378.s003.doc]

Figure S4

**
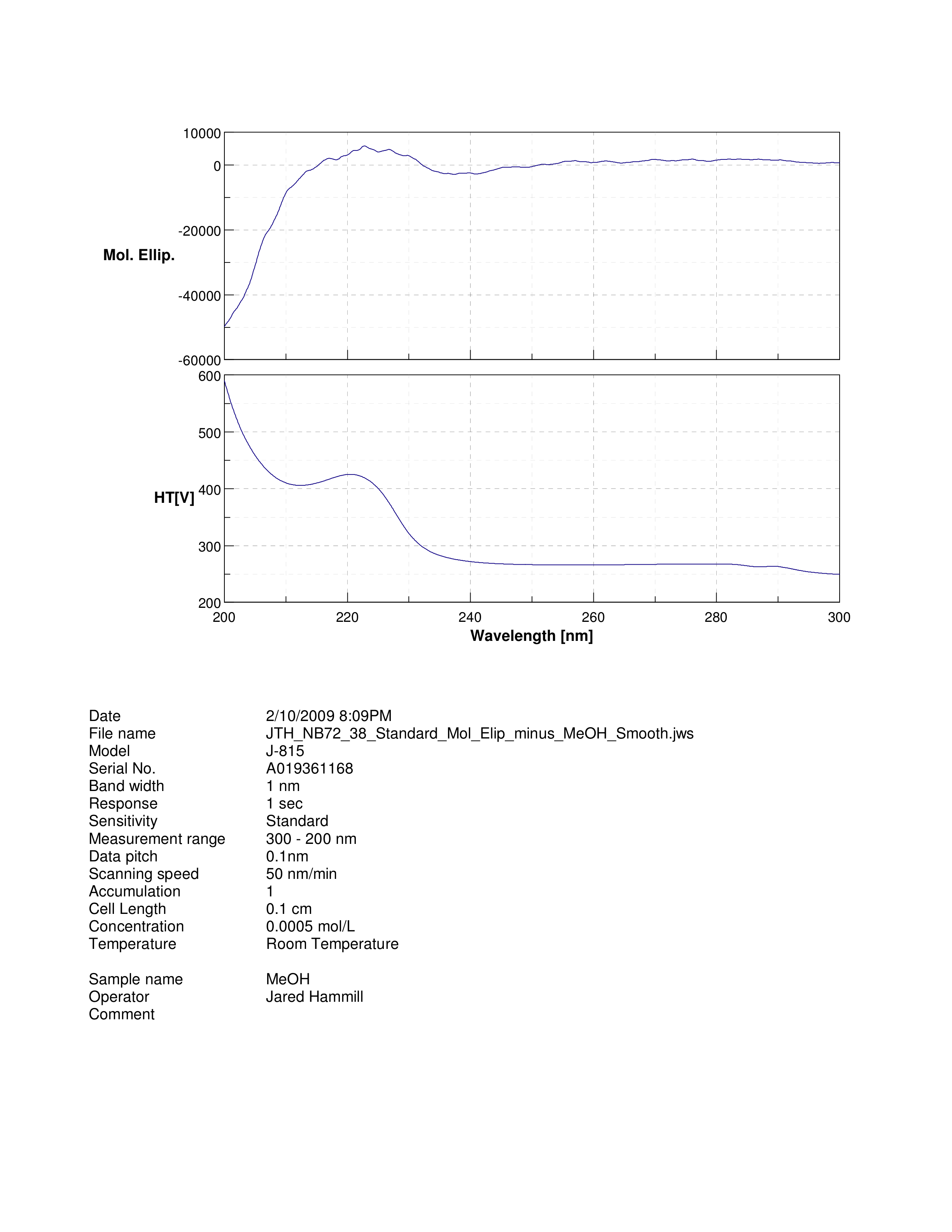
**

Supplement: Figure S4 — CD spectrum of JTH-NB72-38 (0.5 mmol) in MeOH. (0.37 MB DOC) [file pone.0011378.s004.doc]

Figure S5


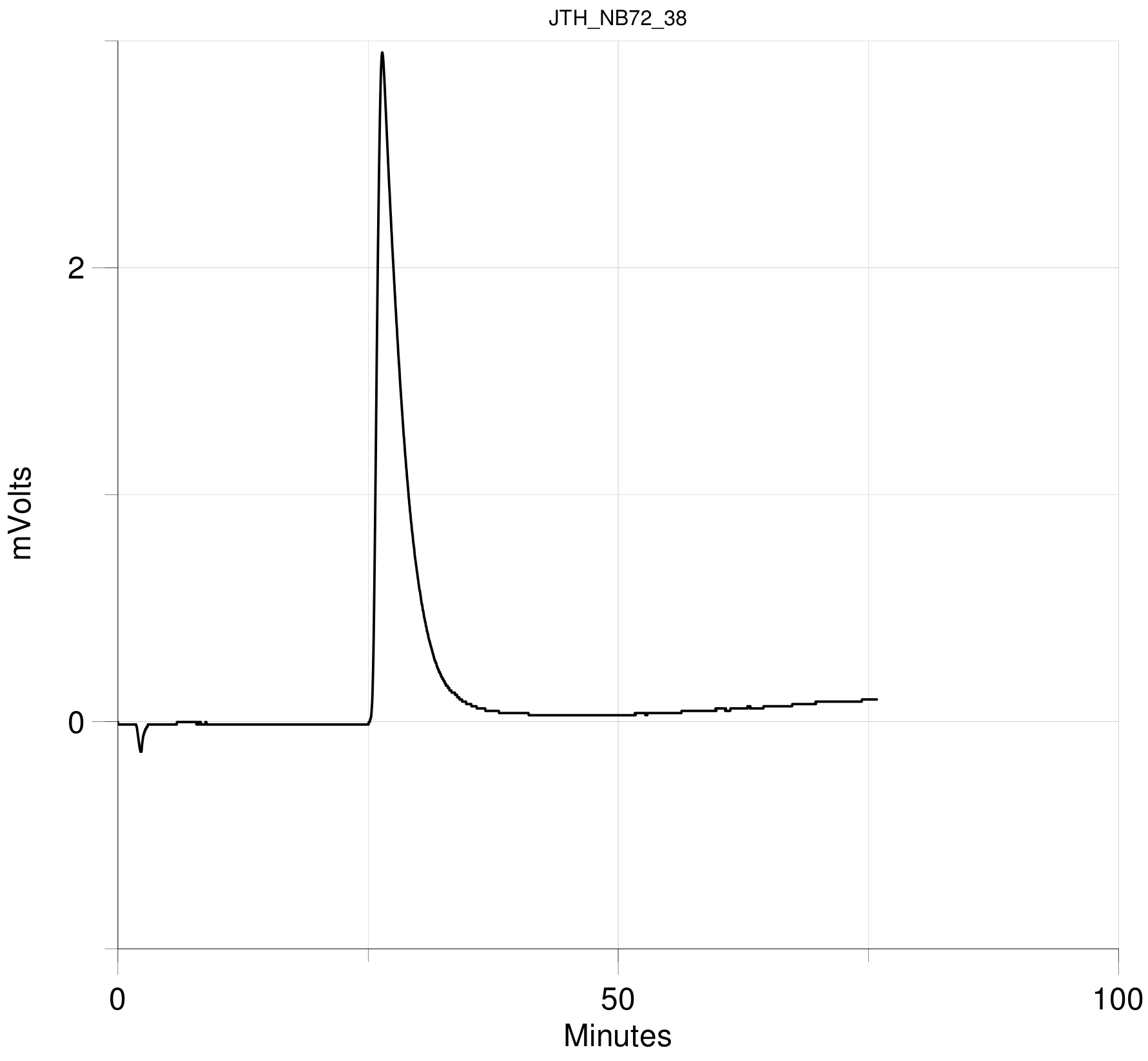

Supplement: Figure S5 — Analytical HPLC trace of JTH-NB72-38 using a linear gradient of 30–100% buffer B in A (A: water containing 0.1% TFA, B: MeOH) over 70 min with UV detection at 220 nm at a flow rate of 0.7 mL/min. (0.14 MB DOC) [file pone.0011378.s005.doc]

Figure S6


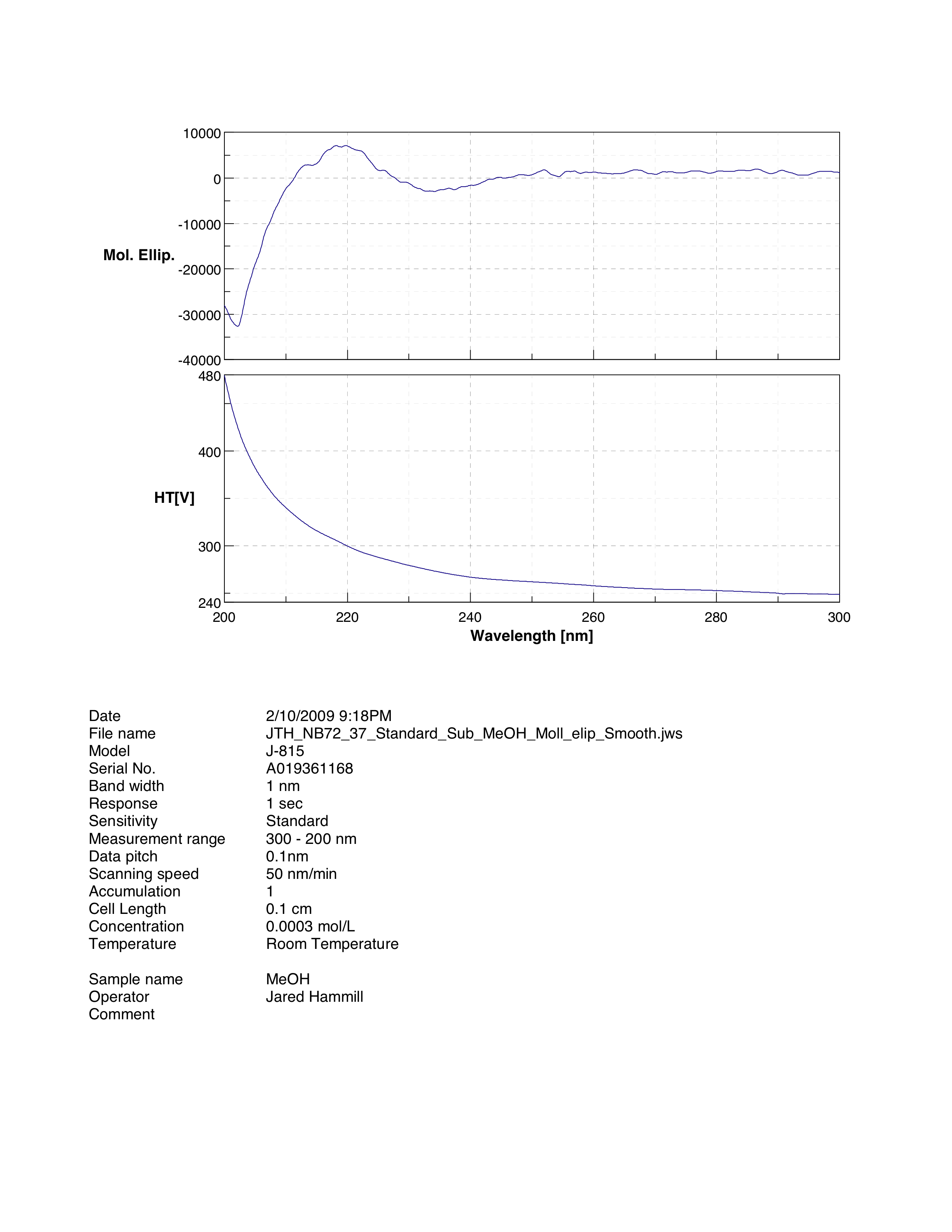

Supplement: Figure S6 — CD spectrum of JTH-NB72-39 (0.5 mmol) in MeOH. (0.37 MB DOC) [file pone.0011378.s006.doc]

Figure S7


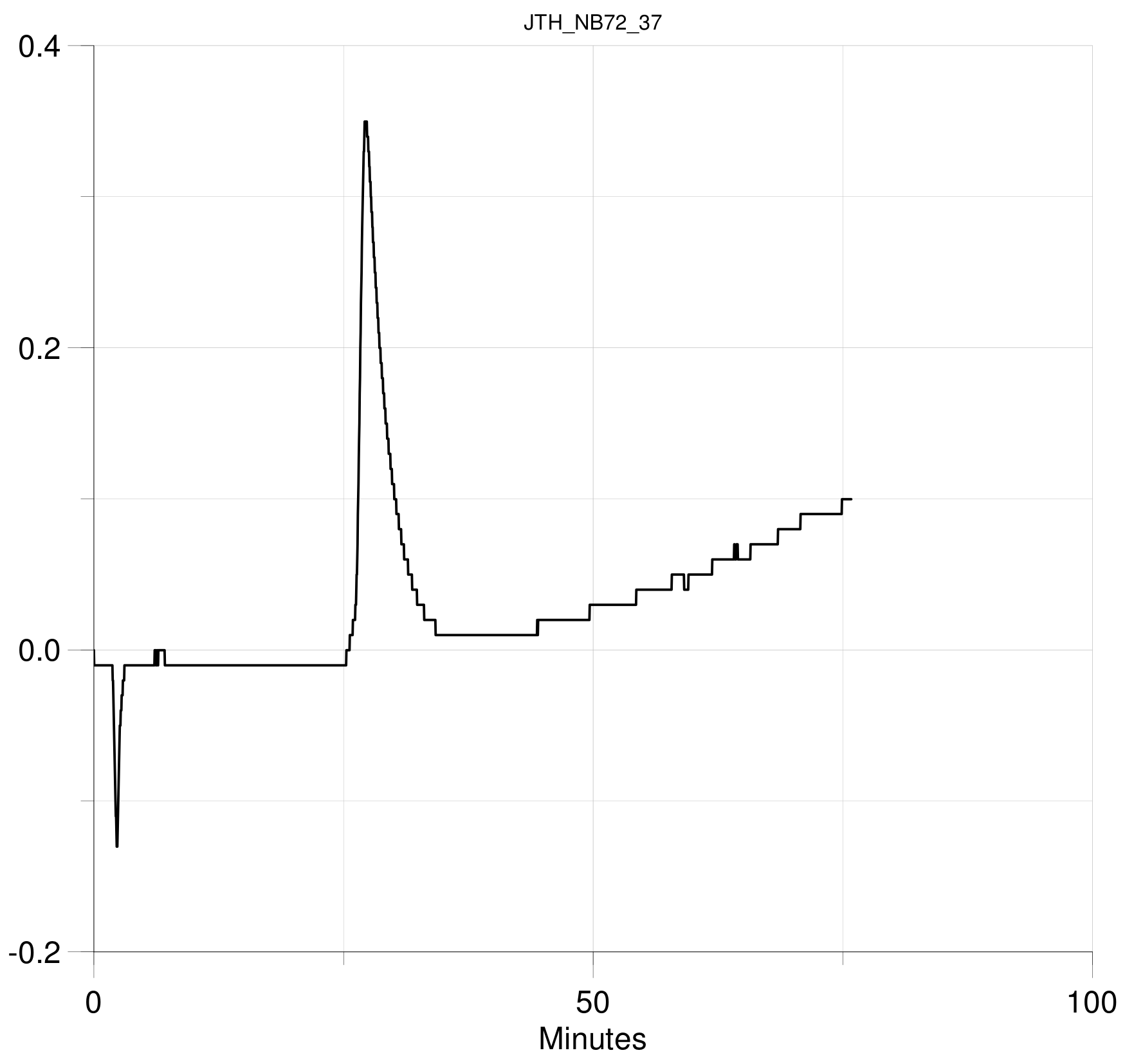

Supplement: Figure S7 — Analytical HPLC trace of JTH-NB72-39 using a linear gradient of 30–100% buffer B in A (A: water containing 0.1% TFA, B: MeOH) over 70 min with UV detection at 220 nm at a flow rate of 0.7 mL/min. (0.14 MB DOC) [file pone.0011378.s007.doc]
