# Supplementary material for: Iterative Structure-Based Peptide-Like Inhibitor Design against the Botulinum Neurotoxin Serotype A
Source: PLoS One. 2010 Jun 30;5(6):e11378. doi: 10.1371/journal.pone.0011378 (PMC2894858; doi:10.1371/journal.pone.0011378)
Supplement: Table S1 — Potencies of structurally characterized BoNT/A LC inhibitors. (0.07 MB DOC) [file pone.0011378.s008.doc]

Table S1

| **Inhibitor** | **Reported Ki** |
| --- | --- |
| QRATKM [39] | 133 mM (IC_50_) |
| RRATKM [39] | 95 mM(IC_50_) |
| RRGC [60] | 157 nM |
| RRGL [60] | 660 nM |
| RRGI [60] | 786 nM |
| RRGM [60] | 845 nM |
| CRATKML [49] | 1.9 mM |
| **I1** [35] | 41 nM |
| **JTH-NB72-39** | 638 nM |
| ArgHX [61] | 60 mM (IC_50_) |
| 2,4-dichlorocinnamic hydroxamate [48] | 300 nM |
| 4-chlorocinnamic hydroxamate [48] | 15 mM (IC_50_) |
|  |  |
|  |  |
|  |  |
|  |  |
