# Supplementary material for: Iterative Structure-Based Peptide-Like Inhibitor Design against the Botulinum Neurotoxin Serotype A
Source: PLoS One. 2010 Jun 30;5(6):e11378. doi: 10.1371/journal.pone.0011378 (PMC2894858; doi:10.1371/journal.pone.0011378)
Supplement: Table S2 — 1H and 13C NMR Data for JTH-NB72-35 (Figure 1) (600 MHz/150 MHz) in D2O (298 K) with MeOH as an internal reference (referenced to 3.34 ppm (1H) and 49.5 ppm (13C)). (0.13 MB DOC) [file pone.0011378.s009.doc]

Table S2.

| Residue # |  | Resonance | ^1^H δ [ppm] | ^13^C  δ [ppm] |
| --- | --- | --- | --- | --- |
|  | Amino Acid (N->C) |  |  |  |
| 1 | Arginine | CO |  | 169.7 |
|  |  | C_α_H | 3.97 (t, *J* = 6.4 Hz, 1 H) | 53.1 |
|  |  | C_β_H | 1.86-1.76 (m, 2 H) | 28.7 |
|  |  | C_γ_H | 1.51-1.42 (m, 2 H) | 23.8 |
|  |  | C_δ_H | 3.03 (dt, *J* = 6.3, 12.7 Hz, 1 H), | 41.0 |
|  |  |  | 3.00 (dt, *J* = 6.3, 12.7 Hz, 1 H) |  |
|  |  | C_ζ_ |  | 157.1 |
| 2 | Arginine | CO |  | 173.3 |
|  |  | C_α_H | 4.35 (t, *J* = 7.2 Hz, 1 H) | 53.8 |
|  |  | C_β_H | 1.72-1.62 (m, 2 H) | 28.9 |
|  |  | C_γ_H | 1.62-1.53 (m, 2 H) | 25.2 |
|  |  | C_δ_H | 3.14 (dt, *J* = 6.7, 13.8 Hz, 1 H), | 41.2 |
|  |  |  | 3.1 (dt, *J* = 6.7, 13.8 Hz, 1 H) |  |
|  |  | C_ζ_ |  | 157.3 |
| 3 | 3-(1-Naphthyl)-alanine | CO |  | 172.7 |
| 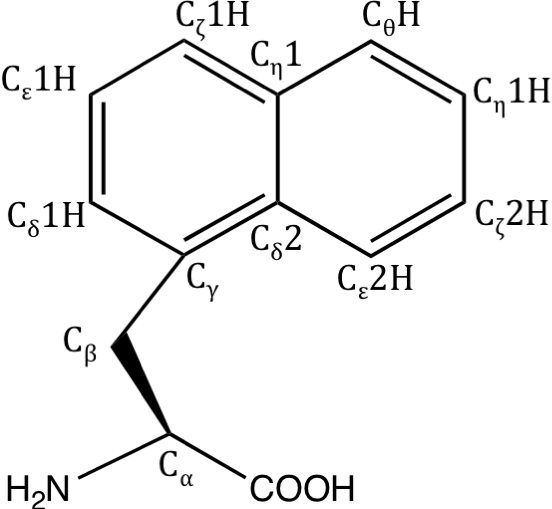 |  | C_α_H | 4.73 (t, *J* = 7.8 Hz, 1 H) | 55.2 |
|  |  | C_β_H | 3.59 (dd, *J* = 7.1, 14.1 Hz, 1 H), | 34.5 |
|  |  |  | 3.50 (dd, *J* = 8.6, 14.1 Hz, 1 H) |  |
|  |  | C_γ_ |  | 132.7 |
|  |  | C_δ_1H | 7.42 (d, *J* = 6.9 Hz, 1 H) | 128.6 |
|  |  | C_δ_2 |  | 132.1 |
|  |  | C_ε_1H | 7.48 (t, *J* = 7.6 Hz, 1 H) | 126.4 |
|  |  | C_ε_2H | 8.15 (d, *J* = 8.5 Hz, 1 H) | 124.0 |
|  |  | C_ζ_1H | 7.86 (d, *J* = 8.2 Hz, 1 H) | 128.5 |
|  |  | C_ζ_2H | 7.63 (t, *J* = 7.6 Hz, 1 H) | 127.3 |
|  |  | C_η_1 |  | 134.2 |
|  |  | C_η_2H | 7.57 (t, *J* = 7.5 Hz, 1 H) | 126.8 |
|  |  | C_θ_H | 7.95 (d, *J* = 8.1 Hz, 1 H) | 129.5 |
