# Supplementary material for: Iterative Structure-Based Peptide-Like Inhibitor Design against the Botulinum Neurotoxin Serotype A
Source: PLoS One. 2010 Jun 30;5(6):e11378. doi: 10.1371/journal.pone.0011378 (PMC2894858; doi:10.1371/journal.pone.0011378)
Supplement: Table S3 — 1H and 13C NMR Data for JTH-NB72-38 (Figure1) (600 MHz/150 MHz) in D2O (298 K) with MeOH as an internal reference (referenced to 3.34 ppm (1H) and 49.5 ppm (13C)). (0.14 MB DOC) [file pone.0011378.s010.doc]

Table S3

| Residue # |  | Resonance | 1H δ ppm | 13C  δ ppm |
| --- | --- | --- | --- | --- |
|  | Amino Acid (N->C) |  |  |  |
| 1 | Arginine | CO |  | 169.8 |
|  |  | C_α_H | 3.99 (t, *J* = 6.5 Hz, 1 H) | 53.1 |
|  |  | C_β_H | 1.88-1.77 (m, 2 H) | 28.7 |
|  |  | C_γ_H | 1.52-1.42 (m, 2 H) | 23.9 |
|  |  | C_δ_H | 3.05 (dt, *J* = 7.1, 13.5 Hz, 1 H), | 41.0 |
|  |  |  | 3.00 (dt, *J* = 7.1, 13.6 Hz, 1 H) |  |
|  |  | C_ζ_ |  | 157.2 |
| 2 | Arginine | CO |  | 173.3 |
|  |  | C_α_H | 4.40 (t, *J* = 7.3 Hz, 1 H) | 53.8 |
|  |  | C_β_H | 1.75-1.64 (m, 2 H) | 28.9 |
|  |  | C_γ_H | 1.52-1.42 (m, 2 H) | 25.1 |
|  |  | C_δ_H | 3.15 (dt, *J* = 6.8, 13.5 Hz, 1 H), | 41.2 |
|  |  |  | 3.11 (dt, *J* = 6.9, 13.5 Hz, 1 H) |  |
|  |  | C_ζ_ |  | 157.3 |
| 3 | Tryptophan | CO |  | 173.2 |
|  | **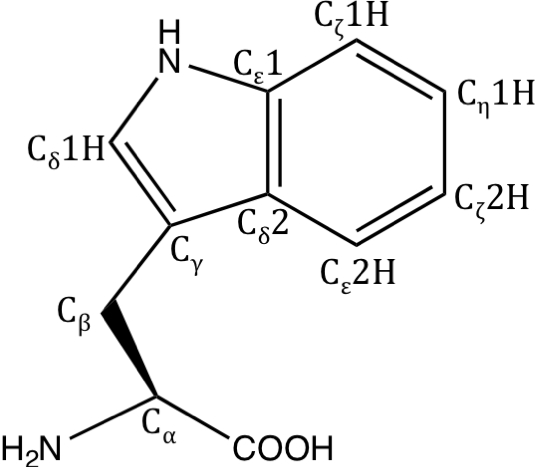** | C_α_H | 4.64 (t, *J* = 7.5 Hz, 1 H) | 55.2 |
|  |  | C_β_H | 3.29 (dd, *J* =7.0, 14.6 Hz, 1 H), | 27.5 |
|  |  |  | 3.22 (dd, *J* = 8.0, 14.7 Hz, 1 H) |  |
|  |  | C_γ_ |  | 109.4 |
|  |  | C_δ_1H | 7.26 (s, 1 H) | 125 |
|  |  | C_δ_2 |  | 127.4 |
|  |  | C_ε_ |  | 136.8 |
|  |  | C_ε_2H | 7.66 (d, *J* = 7.9 Hz, 1 H) | 119.1 |
|  |  | C_ζ_1H | 7.48 (d, *J* = 8.2 Hz, 1 H) | 112.5 |
|  |  | C_ζ_2H | 7.15 (t, *J* = 7.4 Hz, 1 H) | 120.0 |
|  |  | C_η_H | 7.23 (t, *J* = 7.6 Hz, 1 H) | 122.7 |

| 4 | α-aminoisobutyric acid | CO |  | 178.0 |
| --- | --- | --- | --- | --- |
|  |  | C_α_ |  | 57.2 |
|  |  | C_β_H | 1.31 (s, 6 H) | 24.7, 24.5 |
| 5 | Alanine | CO |  | 176.2 |
|  |  | C_α_H | 4.19 (q, *J* = 7.3 Hz, 1 H) | 51.1 |
|  |  | C_β_H | 1.33 (d, *J* = 7.3 Hz, 3 H) | 16.7 |
| 6 | Methionine | CO |  | 174.3 |
|  |  | C_α_H | 4.34 (dd, *J* = 9.3, 5.0 Hz, 1 H) | 53.9 |
|  |  | C_β_H | 2.10-2.0 (m, 2 H) | 30.7 |
|  |  | C_γ_H | 2.60-2.55 (m, 1 H), 2.53-2.49 (m, 1 H) | 30.0 |
|  |  | C_δ_H | 2.05 (s, 3 H) | 14.7 |
| 7 | Leucine | CO |  | 177.8 |
|  |  | C_α_H | 4.28 (dd, *J* = 10.5, 3.9 Hz, 1 H) | 52.9 |
|  |  | C_β_H | 1.75-1.53 (m, 2 H) | 40.2 |
|  |  | C_γ_H | 1.75-1.53 (m, 1 H) | 25.1 |
|  |  | C_δ_H | 0.90 (d, *J* = 5.9 Hz, 3 H), | 22.9, 21.0 |
|  |  |  | 0.84 (d, *J* = 5.9 Hz, 3 H) |  |
