# Supplementary material for: Iterative Structure-Based Peptide-Like Inhibitor Design against the Botulinum Neurotoxin Serotype A
Source: PLoS One. 2010 Jun 30;5(6):e11378. doi: 10.1371/journal.pone.0011378 (PMC2894858; doi:10.1371/journal.pone.0011378)
Supplement: Table S4 — 1H and 13C NMR Data for JTH-NB72-39 (Figure1) (600 MHz/150 MHz) in D2O (298 K) with MeOH as an internal reference (referenced to 3.34 ppm (1H) and 49.5 ppm (13C)). (0.08 MB DOC) [file pone.0011378.s011.doc]

**Table S4**

| Residue # |  | Resonance | ^1^H δ [ppm] | ^13^C  δ [ppm] |
| --- | --- | --- | --- | --- |
|  | Amino Acid (N->C) |  |  |  |
| 1 | Arginine | CO |  | 169.1 |
|  |  | C_α_H | 4.00 (t, *J* = 6.5 Hz, 1 H) | 53.1 |
|  |  | C_β_H | 1.90-1.83 (m, 2 H) | 29.0 |
|  |  | C_γ_H | 1.60-1.47 (m, 2 H) | 24.1 |
|  |  | C_δ_H | 3.16 (t, *J* = 6.8 Hz, 2 H) | 41.1 |
|  |  | C_ζ_ |  | 157.3 |
| 2 | Arginine | CO |  | 173.3 |
|  |  | C_α_H | 4.29 (dd, *J* = 7.5, 10.7 Hz, 1 H) | 53.9 |
|  |  | C_β_H | 1.77-1.65 (m, 2 H) | 28.7 |
|  |  | C_γ_H | 1.60-1.47 (m, 2 H) | 25.1 |
|  |  | C_δ_H | 3.16 (t, *J* = 6.8 Hz, 2 H) | 41.2 |
|  |  | C_ζ_ |  | 157.3 |
| 3 | Phenylalanine | CO |  | 172.8 |
|  |  | C_α_H | 4.55 (t, *J* = 7.6 Hz, 1 H) | 55.7 |
|  |  | C_β_H | 3.11 (dd, *J* = 7.0, 13.9 Hz, 1 H), | 37.5 |
|  |  |  | 3.05 (dd, *J* = 8.3, 13.8 Hz, 1 H) |  |
|  |  | C_γ_ |  | 136.7 |
|  |  | C_δ_H | 7.36 (t, *J* = 7.4 Hz, 2 H) | 129.4 |
|  |  | C_ε_H | 7.28 (d, *J* = 7.5 Hz, 2 H) | 130.0 |
|  |  | C_ζ_H | 7.31 (t, *J* = 7.4 Hz, 1 H) | 127.8 |
| 4 | α-Aminoisobutyric acid | CO |  | 178 |
|  |  | C_α_ |  | 57.3 |
|  |  | C_β_H | 1.35 (s, 3 H), 1.34 (s, 3 H) | 25.0, 24.3 |
| 5 | Alanine | CO |  | 176.1 |
|  |  | C_α_H | 4.22 (q, *J* = 7.2 Hz, 1 H) | 51.0 |
|  |  | C_β_H | 1.37 (d, *J* = 7.3 Hz, 3 H) | 16.8 |

| 6 | Methionine | CO |  | 174.2 |
| --- | --- | --- | --- | --- |
|  |  | C_α_H | 4.39 (dd, *J* = 7.5, 9.3 Hz, 1 H) | 52.9 |
|  |  | C_β_H | 2.18-1.99 (m, 2 H) | 30.1 |
|  |  | C_γ_H | 2.65-2.51 (m, 2 H) | 30.7 |
|  |  | C_δ_H | 2.10 (s, 3 H) | 14.9 |
| 7 | Leucine | CO |  | 177.7 |
|  |  | C_α_H | 4.36 (t, *J* = 7.4 Hz, 1 H) | 53.7 |
|  |  | C_β_H | 1.75-1.59 (m, 2 H) | 40.3 |
|  |  | C_γ_H | 1.75-1.59 (m, 1 H) | 25.0 |
|  |  | C_δ_H | 0.92 (d, *J* = 5.8 Hz, 3 H), | 22.9, 21.0 |
|  |  |  | 0.86 (d, *J* = 5.6 Hz, 3 H) |  |
